# Thermal and oxidative stability of Atlantic salmon oil (*Salmo salar* L.) and complexation with β-cyclodextrin

**DOI:** 10.3762/bjoc.12.20

**Published:** 2016-02-02

**Authors:** Daniel I Hădărugă, Mustafa Ünlüsayin, Alexandra T Gruia, Cristina Birău (Mitroi), Gerlinde Rusu, Nicoleta G Hădărugă

**Affiliations:** 1 Department of Applied Chemistry, Organic and Natural Compounds Engineering, Polytechnic University of Timişoara, Carol Telbisz 6, 300001 Timişoara, Romania; 2 Department of Fish Processing Technology, Akdeniz University, Dumlupinar Boulevard, Campus Antalya, 07058 Antalya, Turkey; 3 Regional Centre for Immunology and Transplant, County Clinical Emergency Hospital Timişoara, Iosif Bulbuca Blvd. 10, 300736 Timişoara, Romania; 4 Department of Food Science, Banat’s University of Agricultural Sciences and Veterinary Medicine “King Mihai I of Romania” - Timişoara, Calea Aradului 119, 300645 Timişoara, Romania

**Keywords:** Atlantic salmon oil (ASO), β-cyclodextrin, differential scanning calorimetry, Karl Fischer titration, omega-3 fatty acid, thermogravimetry

## Abstract

The thermal and oxidative stability of Atlantic salmon oil (*Salmo salar* L.) as well as its β-cyclodextrin (β-CD) complexation ability has been verified for the first time. The main omega-3 fatty acids, EPA and DHA, were significantly degraded, even at 50 °C. Their relative concentrations decrease from 6.1% for EPA and 4.1% for DHA to 1.7% and 1.5% after degradation at 150 °C, respectively. On the other hand, the relative concentrations of monounsaturated and saturated fatty acids remained constant or slightly increased by a few percent after degradation (e.g., from 10.7% to 12.9% for palmitic acid). Co-crystallization of ASO with β-CD at a host–guest ratio of 1:1 and 3:1 from an ethanol–water mixture and kneading methods has been used for the preparation of β-CD/ASO complexes. The analysis of the complexes by thermogravimetry, differential scanning calorimetry (DSC), and Karl Fischer titration (KFT) as well as the decrease of the “strongly-retained” water content confirm the formation of the inclusion compound. Furthermore, the DSC parameters correlate well with the KFT kinetic data for β-CD/ASO complexes.

## Introduction

Functional food products containing omega-3 supplementation are becoming more and more popular and are promoted for their beneficial effects on human health, especially in cardiovascular and brain disorders [1–2] as well as for mental health [3–5]. The main source of omega-3 fatty acid (FA) containing compounds is fish oil [6]. Many fish species are used as the oil source. They are called oily fish and those most used as an omega-3 source are herring, sardines, anchovy, mackerel, and tuna [7]. The fish oil can be separated by various methods such as a wet reduction process, enzymatic or autolytic (silage) processes, dry rendering, solvent or supercritical fluid extraction [7–9]. The most important oily compounds are mono- and especially polyunsaturated FAs (MUFAs and PUFAs, respectively), which appear at a concentration of 63–79.5% in Atlantic salmon (*Salmo salar* L.) oil (ASO) [7,9–11]. The content of omega-3 FAs (especially as glycerides) ranges between 1.3–2.2 g/100 g of salmon [9,12]. The health benefit of omega-3 FAs (but especially EPA, (all-*Z*)-5,8,11,14,17-eicosapentaenoic acid and DHA, (all-*Z*)-docosa-4,7,10,13,16,19-hexaenoic acid) in reducing cardiovascular risk by reducing blood lipids (mainly LDL cholesterol and triglycerides), is well known [13–14].

One of the main disadvantages of PUFAs is their low oxidative and thermal stability. The rate of oxidation of such FAs (especially at higher temperatures) drastically increases with the increasing number of double bonds present, even by few thousand times. For example, the relative oxidation rate of α-linolenic acid (an omega-3 FA) is 2500 times higher in comparison with saturated stearic acid [15]. Light (photo-oxidation) and the presence of heavy metal ions, heme and hemin proteins or superoxide radical anion-generating enzymes are involved in the initiation of auto-oxidation. The main intermediates resulting from the oxidation of FAs are monohydroperoxides, hydroperoxy-epidioxides, as well as peroxy-, alkoxy-, and alkyl radicals [16]. Further thermal degradation or fragmentation of these intermediates leads to odor-active carbonyl compounds such as aldehydes, ketones, alcohols and esters, aldehydic acids, alkanes and alkenes [17]. In the case of fish oil, the main odoriferous compounds resulting from oxidation are propanal, pent-1-en-3-one, hex-3-enal, and pent-1-en-3-ol [18–20].

The stabilization of fish oils can be simply performed by using antioxidants. Natural or synthetic antioxidants are often used. Among natural antioxidants, tocopherols and carotenoids are the most appropriate due to their lipophilic characteristics. Significant suppression of the oxidation process was observed for bulk salmon oil in the presence of α-tocopherol and astaxanthin [17]. Other less lipophilic natural antioxidants are flavonoids, anthocyanins and their glycosides. Huber and collaborators [18] revealed the inhibition of PUFA oxidation by quercetin and its 3-*O*-glucoside. They were as effective as butylated hydroxytoluene (BHT, a synthetic antioxidant) against the oxidation of DHA and methylated linolenic acid (MLN) in emulsion. Anthocyanins (as natural extracts) were effective at increasing the stability of salmon oil when incorporated in a hydroxypropyl methylcellulose matrix for obtaining fish oil packaging films [21]. The addition of BHT, propyl gallate and citric acid to the herring byproducts during the process of fish oil production leads to improved stability up to 400% [22]. Smoking the pink salmon before the oil extraction process was another method for reducing the oxidation by forming phenolic antioxidant compounds [23–24].

Another way to stabilize fish oil is microencapsulation using various matrices. Matrices such as chitosan and *N*-lauroyl chitosan [25], *N*-stearoyl-*O*-butylglycerylchitosan [26], mixtures of soybean soluble polysaccharide and octenyl succinic anhydride [27], hydrolyzed soy protein isolate and maltodextrin [20,28], liposomes [29] and even yeast cell autolysate [30] have been used as shell materials. Lemon oil-based nanoemulsions or fuicodan-containing protein-coated oil-in-water emulsions provide good physical and oxidative stability for the emulsified fish oil [31–32]. Spray granulation, spray drying, and freeze drying methods can be used for obtaining fish oil-containing microcapsules [27,33] and spontaneous emulsification for obtaining nanoemulsions [20,31–32].

One nanoencapsulation method of fish oil components is the molecular encapsulation in cyclodextrins (CDs). The latter are natural or synthetically modified, cyclic oligosaccharides comprising 6, 7, and 8 glucopyranose units for the corresponding α-, β-, and γ-CD types [34–35]. The specific structural architecture of CDs having a hydrophilic exterior and hydrophobic inner cavity allows for tin-containing FA moieties from fish oil [36] or other hydrophobic compounds and mixtures to be more easily encapsulated [37–42]. Thus, the access of oxygen to the reactive center is drastically reduced and the stability of unsaturated fish oil components is enhanced. On the other hand, the water solubility of fish oil components can be enhanced by CD complexation. The combination of CD complexation and microencapsulation of fish oil is also used [43–44]. However, no CD complexation studies on ASO have been published up to now.

The goal of this study was to evaluate the thermal and oxidative stability of ASO (*Salmo salar* L.) and the β-CD complexation by using co-crystallization and kneading methods. The β-CD/ASO complexation has been evaluated by means of thermal and Karl Fischer titration (KFT) methods.

## Results and Discussion

### FA profile of raw and degraded ASO

The quality of the fish oil is strongly related to its FA profile, where PUFAs are the most important FAs. On the other hand, these FAs (and the corresponding glycerides) are easily degraded by oxidation, especially at higher temperatures. An appropriate method for evaluating the overall FA profile is the gas chromatography–mass spectrometry (GC–MS) analysis of the FAs, derivatized to the corresponding methyl esters. The relative concentration of these FA methyl esters (FAMEs) can indicate the stability and/or the degradation level of the ASO in a simple and relevant way. MUFAs were the most concentrated in the raw ASO (Table 1). The highest relative concentration of 35.6% was found for oleic acid methyl ester. On the other hand, PUFAs had a total concentration of 25.4% and the main FAMEs were linoleic acid methyl ester (11.2%), EPA methyl ester (6.1%) and DHA methyl ester (4.1%). Myristic, palmitic and stearic acid methyl esters were the most important saturated fatty acids (SFAs) (3.5%, 10.7% and 2.7%, respectively). Some of the FAMEs could not be clearly identified, even when their MS spectra indicated the class of these compounds. All of them had a relative concentration lower than 0.05%. These results are in good agreement with those obtained by Bencze Rørå and collaborators [23]. They determined a DHA concentration of 4.5% (as methyl ester from the total FAMEs) for the oil from raw fillets obtained from Atlantic salmon, where the diet was supplemented with soybean oil. On the other hand, the concentration of EPA was lower (2.9%). A feeding diet containing various fish and vegetable oils significantly influences the FA profile of ASO. Thus, the EPA and DHA content (as methyl esters) varies in the range of 4.6–6.9% and 6.4–13.6%, respectively [12].

**Table 1:** Relative concentrations of FAs (as methyl esters) obtained from the GC–MS analysis of the derivatized, raw, Atlantic salmon oil (ASO) and those degraded at low (50 °C, code ASO50) and high (150 °C, code ASO150) temperatures.

Entry	Name^a^	KI^b^	Code^c^	Class^d^	Area (ASO) (%)^e^	Area (ASO50) (%)^e^	Area (ASO150) (%)^e^

1	Myristic	1734	C_14:0_	SFA	3.46 ± 0.301	3.34 ± 0.203	3.33 ± 0.727
2	Pentadecanoic	1835	C_15:0_	SFA	0.23 ± 0.012	0.36 ± 0.079	0.43 ± 0.009
3	7,10,13-Hexadecatrienoic	1900	C_16:3_	PUFA	0.31 ± 0.003	0.27 ± 0.045	0.46
4	Palmitoleic	1916	C_16:1_	MUFA	3.50 ± 0.091	3.31 ± 0.215	2.84 ± 0.831
5	Palmitic	1941	C_16:0_	SFA	10.69 ± 0.78	11.35 ± 0.35	12.94 ± 2.26
6	Margaric	2039	C_17:0_	SFA	0.14 ± 0.006	0.20	–
7	Polyunsaturated fatty acid^f^	2096	C_20:4_^#^	PUFA	1.35 ± 0.025	0.78 ± 0.135	–
8	Linoleic	2108	C_18:2_	PUFA	11.20 ± 0.29	11.29 ± 0.11	9.10 ± 2.19
9	Oleic	2121	C_18:1_	MUFA	35.60 ± 1.77	37.64 ± 0.39	35.08 ± 6.85
10	Stearic	2148	C_18:0_	SFA	2.68 ± 0.082	2.79 ± 0.060	3.13 ± 0.645
11	Polyunsaturated fatty acid^f^	2165	C_20:4_^#^	PUFA	0.13 ± 0.038	–	–
12	Monounsaturated fatty acid^f^	2190	C_18:1_^#^	MUFA	0.69 ± 0.402	2.56 ± 0.034	3.09
13	Nonadecanoic	2255	C_19:0_	SFA	–	–	–
14	EPA (5,8,11,14,17-eicosapentaenoic)	2309	C_20:5_	PUFA	6.10 ± 0.378	2.17 ± 0.370	1.74 ± 0.783
15	Polyunsaturated fatty acid^f^	2322	C_20:3_^#^	PUFA	0.84 ± 0.179	0.37	1.16
16	11-Eicosenoic	2347	C_20:1_	MUFA	0.62 ± 0.015	0.48 ± 0.014	–
17	Polyunsaturated fatty acid^f^	2404	C_20:5_^#^	PUFA	–	–	–
18	Polyunsaturated fatty acid^f^	2429	C_20:5_^#^	PUFA	–	–	–
19	Polyunsaturated fatty acid^f^	2523	C_20:5_^#^	PUFA	–	–	–
20	DHA (4,7,10,13,16,19-docosahexaenoic)	2573	C_22:6_	PUFA	4.13 ± 0.731	1.70 ± 0.315	1.45 ± 0.482
21	Polyunsaturated fatty acid^f^	2591	C_20:5_^#^	PUFA	1.37 ± 0.211	–	–
22	Polyunsaturated fatty acid^f^	2709	C_20:5_^#^	PUFA	–	–	–
23	Erucic	2784	C_22:1_	MUFA	1.57	2.45 ± 0.322	1.73 ± 0.445
24	Behenic	2855	C_22:0_	SFA	–	–	–
25	Nervotic	3173	C_24:1_	MUFA	–	–	–
26	Other minor compounds^g^				15.39	18.94	23.52

	Total quantified FAs (%)				84.61	81.06	76.48
	Total SFAs (%)				17.2	18.04	19.83
	Total MUFAs (%)				41.98	46.44	42.74
	Total PUFAs (%)				25.43	16.58	13.91

^a^The FAME name; ^b^Kovats index (calculated according to C_8_–C_20_ alkane standard solution GC data; higher KI values were obtained by extrapolation); ^c^the corresponding FA code (C_x:y_ represents the fatty acid containing “x” carbon atoms and “y” double bonds; ^d^SFA – saturated fatty acid, MUFA – monounsaturated fatty acid, PUFA – polyunsaturated fatty acid; ^e^the FAME concentration, calculated as the percent ratio between the GC peak area of the compound and the sum of all GC peak areas; ^f^isomers are indicated by “^#^” (using only the MS data and not the standard FAMEs); ^g^the other minor compounds were FAMEs having concentrations lower than 0.05% or they do not belong to this class (e.g., aldehydes).

The high content of MUFAs and PUFAs in the ASO (42% and 25.4%, respectively) is correlated to a higher susceptibility to oxidation, especially at elevated temperatures. Consequently, two degradation temperatures named low (50 ± 1 °C) and high (150 ± 1 °C) have been proposed. The total FA content decreases after degradation at high temperature (from 84.6% to 76.5%, Table 1). This variation is especially due to the degradation of the PUFAs (as glycerides or free FAs). The total PUFA content decreases from 25.4% in raw ASO to 16.6% and 13.9% for fish oil degraded at low and high degradation temperatures, respectively. On the contrary, the total SFA content increases from 17.2% to 19.8%. This fact is due to the higher oxidative stability of the SFAs, even the MUFAs had higher stability in comparison with PUFAs by means of the total content. Unfortunately, they are isomerized to the corresponding “bad” *trans*-diastereoisomers at high degradation temperatures, without modifying the structural class [36]. More interesting is the variation of the individual FAs (such as methyl ester) with the degradation temperature. While the myristic acid content was almost constant in the FAME mixtures (3.3–3.5%), an increase of the relative concentrations of palmitic and stearic acids was observed (from 10.7% to 12.9% and from 2.7% to 3.1%, respectively; Table 1). On the other hand, the most significant omega-3 fatty acids, EPA and DHA, are easily degraded even at low temperatures. Thus, the relative concentration of the EPA methyl ester decreases from 6.1% to 2.2% after oxidative degradation at 50 °C and to 1.7% after degradation at high temperature. A similar behavior was observed for the case of DHA (from 4.1% to 1.7% and 1.45%, respectively). This observation is in good agreement with the energy requirement for H-atom abstraction in the oxidation process. It varies from 422 kJ·mol^−1^ for the case of a terminal methyl and 322 kJ·mol^−1^ for a single allyl group (for MUFAs and their glycerides) to 272 kJ·mol^−1^ for the C–H disruption in the case of a methylene group of a 1,4-pentadiene system (which often appear in the PUFAs and their corresponding glycerides) [15]. This thermodynamic behavior increases the oxidation rate of α-linolenic acid (an omega-3 FA) by 2500 times in comparison with the corresponding saturated compound, i.e., stearic acid. Furthermore, it was observed that the concentration of conjugated dienes (not counted as PUFAs) remarkably increased after oxidative degradation of fish oil [17,21]. These studies also support our results on thermal and oxidative degradation of ASO at low and high temperature degradation conditions.

### Preparation and analysis of β-CD/ASO complexes

β-CD/ASO complexes were obtained by using two different methods: co-crystallization from an ethanol–water mixture and by kneading techniques. No literature data on β-CD/ASO complexes have been reported yet. The reason for choosing the controlled crystallization of β-CD/ASO complexes was related to the possibility to attain the equilibrium between the noncomplexed and complexed fish oil components and to obtain a more “pure” complex. The crystals will be mainly formed by CD/bioactive compound complex than by noncomplexed components (CD and FA glycerides). Such a complex is easier to characterize, even if the total recovered yield is low. On the other hand, the kneading method provides CD complexes at a higher recovered yield, but there is a nonuniform mixture of noncomplexed and complexed CD and fish oil components. Thus, the recovered yield (calculated as the percent ratio between the mass of the dried complex and the sum of the ASO and hydrated β-CD masses) was 39.6 ± 0.03% and 58.30 ± 9.81% for the β-CD/ASO complex at a molar ratio of 1:1, obtained by co-crystallization and kneading methods, respectively. For the preparation of the β-CD/ASO complex at a molar ratio of 3:1, the corresponding recovered yields were 75.13 ± 1.66% and 79.15 ± 4.64%, respectively. The recovered yield of the β-CD/ASO complex is significantly lower for the 1:1 molar ratio in comparison with the 3:1 molar ratio. However, these values were higher for the kneading method. These results can be explained by the molecular encapsulation of FA glycerides by CDs, which theoretically implies a 3:1 molecular ratio (further studies are needed). A high content of fish oil remains nonencapsulated. It is washed with ethanol during the separation process for the 1:1 molar ratio.

The analysis of the β-CD/ASO complexes was focused on the evaluation of the water content and the type of water molecules (i.e., the behavior of the water during the various analyses). This approach provided the main assessment of the molecular encapsulation process.

### Thermogravimetric (TG) analysis

**Figure 1:**
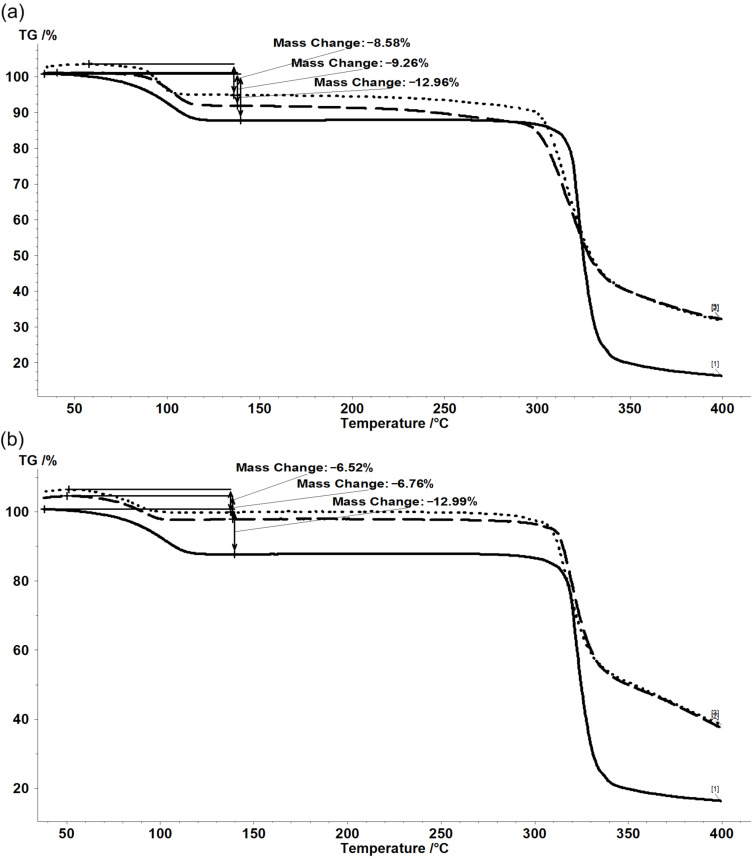
Superimposed thermograms for β-CD (solid line) and (a) β-CD/ASO_1:1_a&b (duplicate) or (b) β-CD/ASO_3:1_a&b (duplicate). For sample name abbreviations, see Table 3.

TG analysis of β-CD/ASO complexes can furnish information on the behavior during heating. Both β-CD and its complexes release water (and possibly other solvents used in the complexation process) up to ≈140 °C, but mainly up to 100 °C. ASO components are nonvolatile and the mass variation up to 250 °C is very low and almost null for β-CD. After this temperature, the degradation of β-CD as well as the fish oil components occurs. The mass loss for the first temperature interval is almost 13% for β-CD, while for complexes it was 8.93 ± 0.50% and 6.62 ± 0.17% for the 1:1 and 3:1 molar ratio using the co-crystallization method, respectively. These values were more similar in the case of the kneading method (7.00 ± 0.46% and 6.74 ± 1.15%, respectively) (Figure 1). On the other hand, the mass loss up to the start of the β-CD degradation is significant for complexes obtained by the co-crystallization method at a 1:1 molar ratio (1.8 ± 0.54%, see Supporting Information File 1 for details). This can be due to the release of “strongly-retained” water and other solvents at higher temperatures. One of the most important parts is the release of water from complexes. According to the TG analysis, the water content is 4.1–6.4% lower than for the β-CD. This observation supports the formation of the inclusion compound by partial replacement of the hydration water from the CD cavity by the guest compounds (FA glycerides). Studies on the evaluation of water and solvent contents in host–guest supramolecular systems by thermal analyses have already been performed for CD/flavonoid and CD/essential oil complexes [37,39].

### Differential scanning calorimetry (DSC) analysis

**Figure 2:**
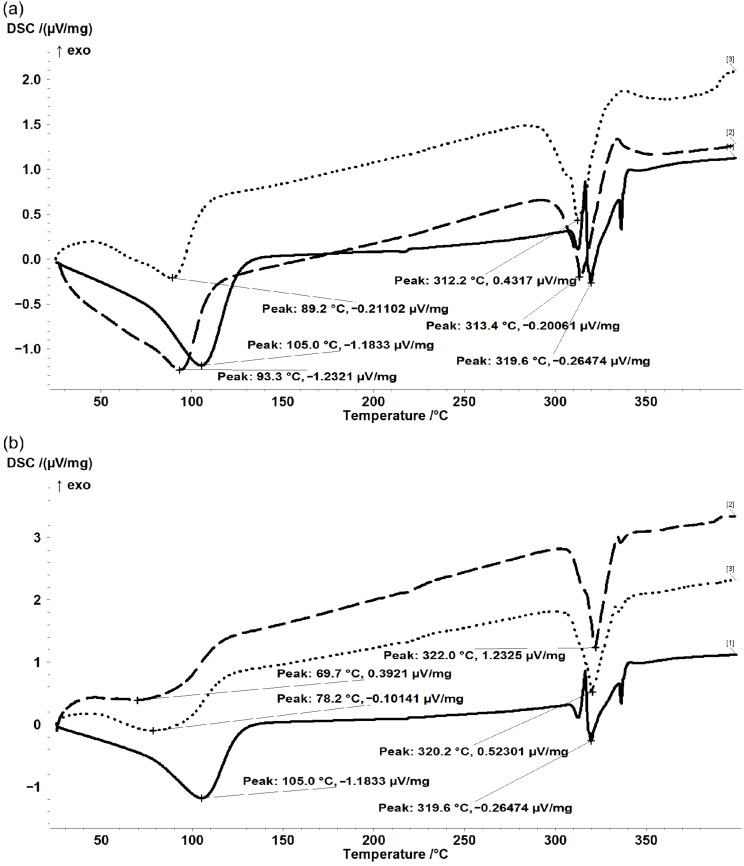
Superimposed DSC data for β-CD (solid line) and (a) β-CD/ASO_3:1_a&b (duplicate) or (b) β-CD/ASO_3:1(k)_a&b (duplicate). For sample name abbreviations, see Table 3.

DSC can provide further information on the physical and chemical processes occurring during heating, even if the guest compound is not volatile up to the CD degradation temperature. Commercial β-CD has two important DSC peaks corresponding to the release of both “surface” and “strongly-retained” water molecules at 105 °C and to the β-CD decomposition at 319.6 °C (Figure 2). The decomposition of β-CD from its fish oil complexes also occurs in the temperature range close to noncomplexed β-CD, especially for the kneading method (319.3 ± 2.9 °C) (see Supporting Information File 1 for all information related to the DSC analysis). One of the most important DSC peaks is that related to water release. It was evident that the 1:1 molar ratio is not appropriate for molecular encapsulation of ASO glycerides in β-CD because the DSC peak corresponding to water release appears at similar or even higher temperature values (103 ± 5 °C and 121 ± 4 °C for co-crystallization and kneading methods, respectively, Figure 2). These values also suggest a significant content of “strongly-retained” water in the complex, which can be due to the presence of noncomplexed or not completely complexed β-CD. The differences for the case of 3:1 molar ratio complexes are evident. The DSC peak temperatures corresponding to water release decreases to 91.3 ± 2.9 °C and 74 ± 6.0 °C for the co-crystallization and kneading methods, respectively (Figure 2 and Supporting Information File 1). The decrease of these DSC peak temperatures can be explained by the formation of the host–guest molecular inclusion compound that allows replacement of the “strongly-retained” water molecules inside the cavity by the hydrophobic FA moieties from ASO. The β-CD/ASO complex mainly contains “surface” water molecules that are easier to be released [45]. On the other hand, the calorimetric effect corresponding to water release is strongly reduced after complexation from 279.6 μV·s·mg^−1^ to 129.2 ± 14.9 μV·s·mg^−1^ for the co-crystallization method and 101.3 ± 17.3 μV·s·mg^−1^ for the kneading method. This means that the total water content is lower and/or more weakly physically bound in the β-CD/ASO complex structure in comparison with the case of noncomplexed β-CD. However, the quantification of the total “surface” and “strongly-retained” water content is very difficult by thermal methods.

### Karl Fischer titration (KFT) analysis

KFT analysis can provide more accurate results on the water content and release from β-CD/ASO complexes. The advantages of this chemical method in comparison with other classical methods (e.g., oven drying or similar drying methods) is related to the selectivity for water (no other compounds such as solvents or volatiles will be detected), the controllable analysis temperature (room temperature or a preset lower/higher temperature can be used), and the diffusion of water to the surface of the CD complex particle, which is enhanced by the possibility to control the hydrophobicity of the solvent mixture used for KFT analysis [45–50].

The total water content of 13.7 ± 0.2% for β-CD as determined by KFT analysis (Table 2) is higher than the TG mass loss corresponding to the water release, even up to 140 °C (≈13%). The water content of β-CD/ASO complexes is significantly lower, especially for the case of the co-crystallization method (7.30 ± 0.27% and 8.96 ± 0.15% for 1:1 and 3:1 molar ratio, respectively). The variation in the total water content of the β-CD/ASO complexes obtained by the kneading method is similar, but the values are higher (8.60 ± 0.41% and 11.60 ± 0.34%, respectively). The KFT results are in agreement with the TG data on the dissimilarity between noncomplexed and complexed β-CD. On the other hand, the overall water content is higher than the TG mass loss that corresponds to the water (or other solvent) release from the β-CD and its fish oil complexes. A higher water content for the complexes obtained at a 3:1 molar ratio by using both the co-crystallization and kneading methods has been observed (difference of 1.7–3% in comparison with the complexes obtained at 1:1 molar ratios; see Table 2). It is obvious that a higher molar content on β-CD in the complex leads to a higher content of water, especially for the case of the kneading method. This observation also agreed with the recovered yield of β-CD/ASO complexes (yield of 39.6% and 58.3% / water content of 7.3% and 9% for 1:1 molar ratio and recovery yield of 75.1% and 79.2% / water content of 8.6% and 11.6% for 3:1 molar ratio).

**Table 2:** Water content and mean water reaction rates, ν, in the main time ranges (10–30 s and 30–90 s) of β-CD/ASO complexes and commercial β-CD obtained by the KFT method.

Entry	Code	Water content (%)	“Surface” water reaction rate, ν_1_ (mM·s^−1^)^a^	“Strongly-retained” water reaction rate, ν_2_ (mM·s^−1^)^a^

1	β-CD	13.69 ± 0.21^b^	0.445 ± 0.136	0.055 ± 0.017
2	β-CD/ASO_1:1_a	7.49 ± 0.67^c^	0.039 ± 0.006	0.025 ± 0.002
3	β-CD/ASO_1:1_b	7.11 ± 0.57^d^	0.034 ± 0.003	0.021 ± 0.002
4	β-CD/ASO_3:1_a	9.06 ± 0.22^e^	0.109 ± 0.011	0.036 ± 0.005
5	β-CD/ASO_3:1_b	8.85 ± 0.25^d^	0.124 ± 0.026	0.033 ± 0.004
6	β-CD/ASO_1:1(k)_a	8.88 ± 0.29^c^	0.146 ± 0.021	0.034 ± 0.006
7	β-CD/ASO_1:1(k)_b	8.31 ± 0.25^d^	0.049 ± 0.011	0.026 ± 0.004
8	β-CD/ASO_3:1(k)_a	11.84 ± 0.19^d^	0.198 ± 0.020	0.021 ± 0.003
9	β-CD/ASO_3:1(k)_b	11.36 ± 0.26^c^	0.181 ± 0.034	0.053 ± 0.006

^a^ν_1_ and ν_2_ represent the mean water reaction rates from the KFT analysis for the time intervals corresponding to “surface” water (10–30 s) and “strongly-retained” water molecules (30–90 s), respectively; the number of replicates were ^b^eight, ^c^six, ^d^seven and ^e^five.

**Figure 3:**
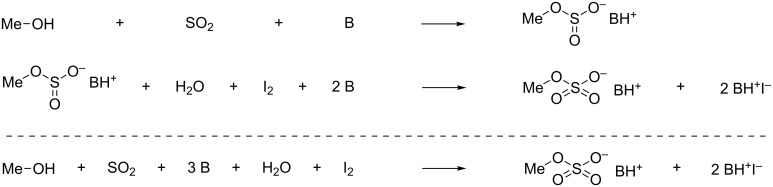
The equation of the KF chemical reaction.

**Figure 4:**
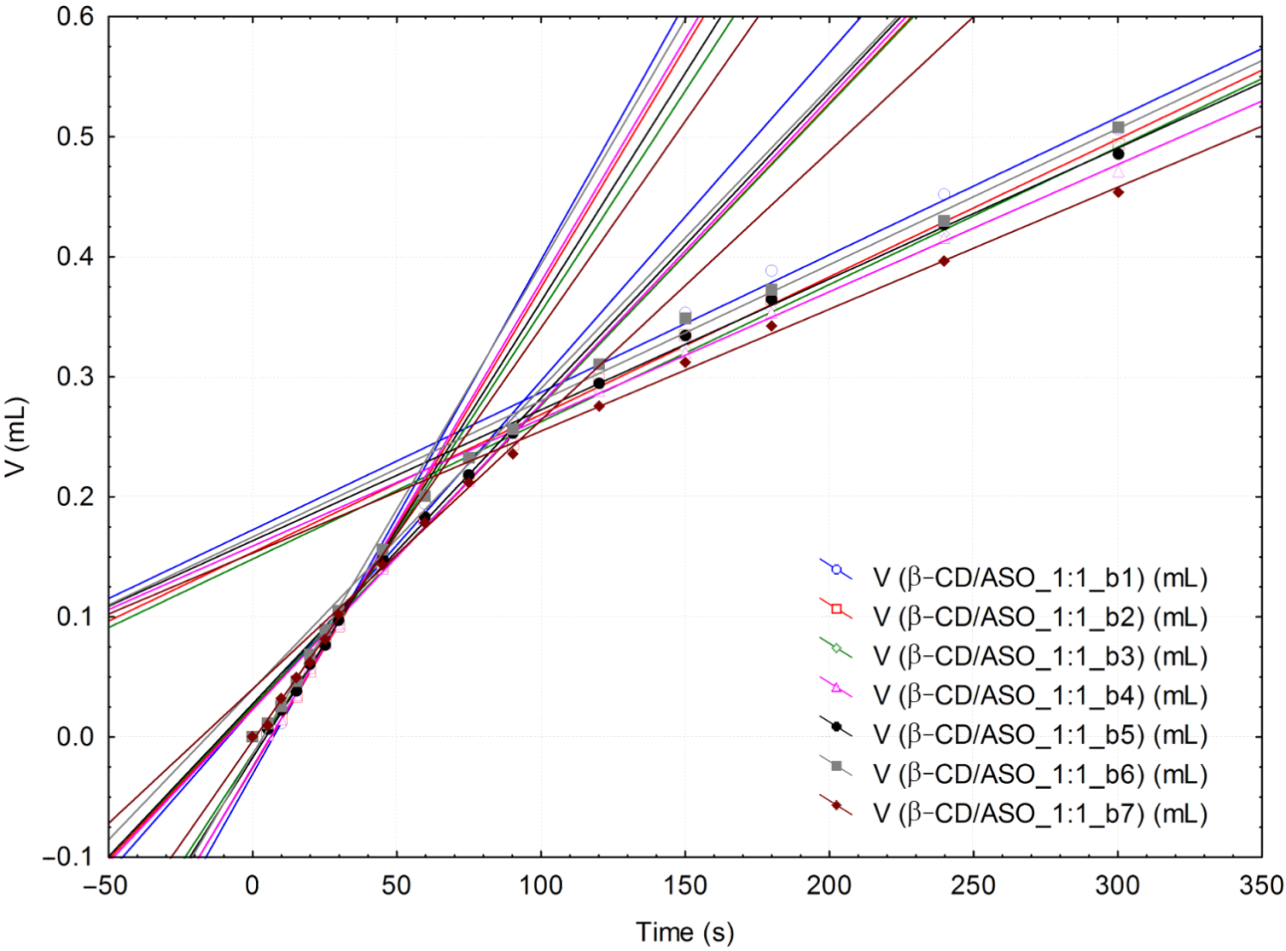
Superimposed volume versus time linear correlations (all three specific intervals) from the KFT analysis of β-CD/ASO_1:1 (only the second replicate “b” of the complex is presented; there are seven determinations).

**Figure 5:**
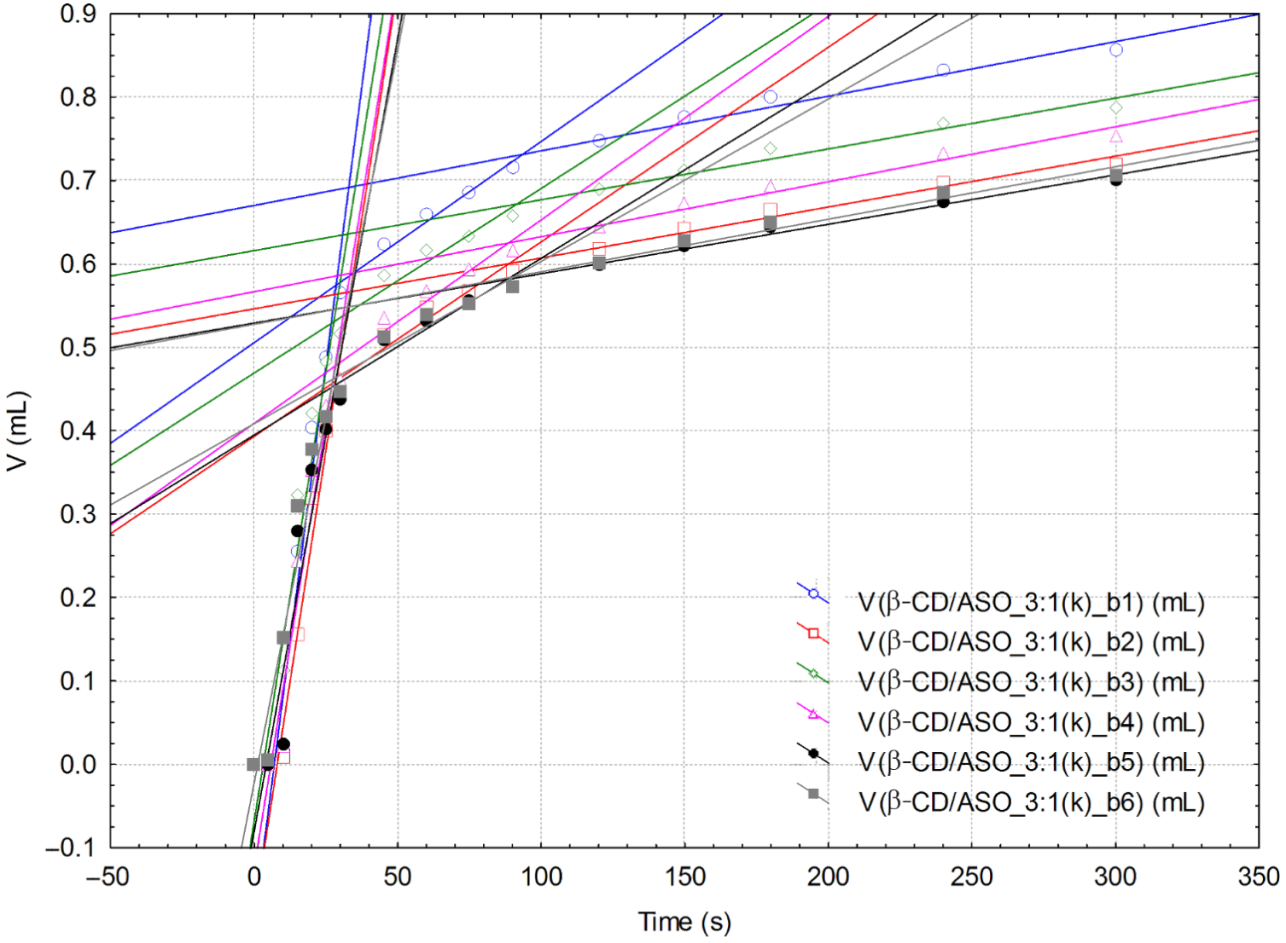
Superimposed volume versus time linear correlations (all three specific intervals) from the KFT analysis of β-CD/ASO_3:1(k) (only the second replicate “b” of the complex is presented; there are six determinations).

KFT is a valuable technique for evaluating the diffusion of water molecules inside the β-CD/ASO complex particles that are not soluble in the KFT working medium (generally methanol). Thus, the variation of the titration volume in time can be correlated to the variation of the water consumption in time (or variation of water concentration; see Figure 3 for the KF reaction, where “B” represents an organic base) by knowing the volume of the reaction medium (which was 30 mL and increases during titration by a maximum of 4 mL; this was also accounted for) and the titer of the iodine solution (4.9447 ± 0.1759 mg H_2_O·mL^−1^). Three important pseudo-linear ranges on the KFT titration volume versus time plots can be observed (Figure 4 and Figure 5; see also Supporting Information File 1). In the first range of ≈10–30 s the water very rapidly reacts and was considered as “surface” water [47]. The second interval corresponds to the reaction of water molecules that slowly diffuse from the inside of the complex particles (so-called “strongly-retained” water molecules) and the pseudo-linear time range is ≈30–90 s. The last interval is related to the “normal” drift of the KFT process (possible some of the “strongly-retained” water molecules can be titrated during this interval). Consequently, the variation of the water consumption over the specified time intervals represents the mean water reaction rate, *v*. Therefore by comparing the values of *v* for different β-CD/ASO complexes and β-CD, it is possible to evaluate the success of the molecular encapsulation process. The main difference between β-CD and its complexes for the “surface” water was observed (Table 2). The corresponding water reaction rate decreases from 0.45 mM·s^−1^ for commercial β-CD to 0.04–0.2 mM·s^−1^ for the corresponding ASO complexes. Complexes obtained by the kneading method had higher “surface” water reaction rates than those obtained by the co-crystallization method. Furthermore, complexes obtained by using a 3:1 molar ratio revealed higher “surface” water reaction rates. On the other hand, the “strongly-retained” water reaction rates for complexes had lower values, especially for products obtained by co-crystallization at a 1:1 molar ratio (Table 2). The results of the KFT kinetics indicate that the diffusion of “surface” water is lower in complexes than in commercial β-CD particles and even up to ten times slower for complexes obtained at a 1:1 molar ratio. The diffusion of “strongly-retained” water in complexes is similar to the case of commercial β-CD. Therefore, the hydrated β-CD/ASO complexes have a relatively low content of “surface” water that can be easily released, while the “strongly-retained” water molecules have similar behavior similar to β-CD. However, the overall water content is drastically reduced after complexation and the ratio between the volumes of the titrant corresponding to “surface” and “strongly-retained” water can furnish further information on the molecular encapsulation efficiency (further studies are needed).

### Thermal analysis – KFT correlations

Thermal analysis methods such as TG and DSC provide information on the releases of volatile compounds and on the energetic effect of these processes (as well as other ones), but no differentiation between compounds can be established. On the other hand, KFT provides a selective determination for water. Consequently, the correlation between the thermal and KFT water/volatile-related parameters can indicate the accuracy of the analyses and the performance of the molecular inclusion process.

Generally, the differences between values of the thermal and KFT parameters for noncomplexed β-CD and β-CD/ASO complexes are significant and the correlation equations lead to valuable correlation coefficients. However, the other statistical parameters for such correlational equations are not appropriate because β-CD and the group of complexes provide a statistically nonsignificant correlation. Consequently, the statistically valuable correlation equations between thermal and KFT water-related parameters in the β-CD/ASO complex class (without β-CD) were obtained.

Statistically significant correlation equations between DSC peak parameters corresponding to water/volatile compound (solvents) release and KFT parameters for β-CD/ASO complexes have been obtained. It is evident that the DSC peak temperature corresponding to water release (*Peak*_(DSC-Water)_ parameter) increases with the decrease of the total KFT water content (*Water*_(KFT)_ parameter) or the “surface” water reaction rate (*v*_1(KFT)_ parameter) (Equation 1 and Equation 2). This observation supports the fact that a high content of “surface” water in β-CD complexes leads to a lower value for the corresponding DSC peak (“surface” water is released at lower temperature). Moreover, the “surface” water that corresponds to the lower DSC peak temperature results in a higher water reaction rate.

[1]
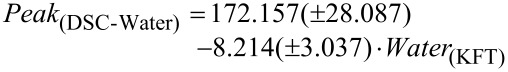


*n* = 8, *r* = 0.741, *s* = 13.6, *F* = 7.3

[2]
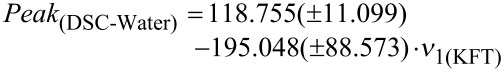


*n* = 8, *r* = 0.669, *s* = 15.0, *F* = 4.8

The finding from the DSC–KFT correlations is also supported by the correlational equation between the total water content of β-CD/ASO complexes, *Water*_(KFT)_, and the mean water reaction rate, *v*_1(KFT)_ (Equation 3). Statistically significant equations were obtained even when β-CD is included in analysis (Equation 4).

[3]
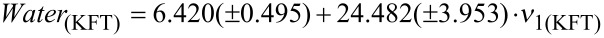


*n* = 8, *r^2^* = 0.930, *s* = 0.67, *F* = 38.4

[4]
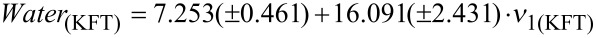


*n* = 9, *r* = 0.929, *s* = 0.87, *F* = 43.8

**Table 3:** Abbreviations used.

Abbreviation	Full text

ANOVA	analysis of variance
ASO	Atlantic salmon oil
β-CD	β-cyclodextrin
β-CD/ASO_1:1_a&b	β-cyclodextrin/Atlantic salmon oil complex obtained by co-crystallization at a 1:1 molar ratio (duplicates “a” and “b”)
β-CD/ASO_1:1(k)_a&b	β-cyclodextrin/Atlantic salmon oil complex obtained by kneading at a 1:1 molar ratio (duplicates “a” and “b”)
β-CD/ASO_3:1_a&b	β-cyclodextrin/Atlantic salmon oil complex obtained by co-crystallization at a 3:1 molar ratio (duplicates “a” and “b”)
β-CD/ASO_3:1(k)_a&b	β-cyclodextrin/Atlantic salmon oil complex obtained by kneading at a 3:1 molar ratio (duplicates “a” and “b”)
BHT	butylated hydroxytoluene
DHA	(all-*Z*)-docosa-4,7,10,13,16,19-hexaenoic acid
DSC	differential scanning calorimetry
EPA	(all-*Z*)-5,8,11,14,17-eicosapentaenoic acid
FA	fatty acid
FAME	fatty acid methyl ester
GC–MS	gas chromatography–mass spectrometry
KFT	Karl Fischer titration
KI	Kovats index
LDL	low-density lipoprotein
MLN	methylated linolenic acid
MUFA	monounsaturated fatty acid
PUFA	polyunsaturated fatty acid
SFA	saturated fatty acid
TG	thermogravimetry

## Conclusion

ASO is very unstable even at low degradation temperature. The relative concentration of the primary and most valuable compounds, EPA and DHA, is strongly reduced after thermal and oxidative degradation. It is obvious that protection against oxidation for these omega-3 fatty acid glycerides is needed. Thus, good yields from preparation of β-CD/ASO complexes by co-crystallization and kneading have been obtained. Furthermore, thermal and KFT analyses support the conclusion of formation of the β-CD/FA glyceride inclusion complex by means of water behavior. Hydration water is easier released from the β-CD/ASO complexes obtained at a 3:1 molar ratio. Both DSC and KFT analyses demonstrate this finding with a lower peak temperature and lower “strongly-retained” water reaction rate, respectively. The total water content (evaluated by both TG and KFT analyses) and the DSC calorimetric effect related to water release are significantly lower in the case of β-CD/ASO complexes in comparison with commercial β-CD. All these analyses performed for the first time for the stabilized β-CD/ASO complexes confirm the formation of the inclusion compound.

## Experimental

### Materials

Atlantic salmon (*Salmo salar* L.) was obtained from the local market (Timişoara, Romania) as a raw product in the spring of 2014. It was an aquaculture product of Norwegian origin. Only the meaty fish parts were used for oil extraction. GC-grade hexane (Sigma-Aldrich) was the main solvent used for raw and degraded fish oil dilutions. The Supelco 37 Component FAME mix (Sigma-Aldrich) and C_8_–C_20_ alkane standard solution (Fluka Chemie AG) were the main tools for identifying the FAMEs in derivatized raw and degraded fish oil. Anhydrous sodium sulfate (p.a., Merck & Co.) was used for drying the raw and degraded fish oil solutions. β-CD hydrate (>98%) from CycloLab (Budapest, Hungary) was used for ASO molecular encapsulation. Ethanol 96% (v/v, Chimopar, Bucharest, Romania) was used for β-CD complexation of ASO. A boron trifluoride–methanol complex (20%, Merck & Co., Inc.) was used for FA derivatization. Finally, Hydranal-Titrant 5, Hydranal-Solvent and Hydranal-Water Standard 10.0 (Sigma-Aldrich, Buchs, Switzerland) were used for the KFT water analysis of β-CD/ASO complexes.

### ASO extraction

The extraction of raw ASO was performed by a cooking and pressing method. The salmon was boiled for 20 min at a fish/water ratio of 1:2 at 112–114 °C under a pressure of 1.5–1.6 atm in a 5 L aluminum reactor. The crude extract was cooled and filtered, while the fish residue was pressed in a strainer-pressing tool, and the raw oil was separated from the aqueous layer. The raw ASO was centrifuged for 20 min at 9600*g* and 4 °C. The obtained clear ASO was stored at 4 °C in the refrigerator until use.

### Degradation of ASO

The ASO was degraded under thermal and oxidative conditions. The degradation was performed in a temperature-controlled oven (Nabertherm L1/12, Lilienthal, Germany) in air at atmospheric pressure (70% relative humidity). The ASO was uniformly distributed on the bottom of the glass flask. Approximately 50 mg of fish oil were needed to obtain a thin film on a plate surface of 315 cm^2^. Two degradation temperatures were chosen: a low temperature of 50 °C and a high temperature of 150 °C. The degradation time was 2 h. After the degradation reaction and cooling of the samples, the degradation products were extracted with 4 mL of hexane. The solution was dried over anhydrous sodium sulfate and subjected to derivatization.

### Preparation of β-CD/ASO complexes

β-CD/ASO complexes were prepared by two methods: (1) the co-crystallization from an ethanol–water mixture, which allows the equilibrium between the complexed and noncomplexed fish oil components to be achieved, and (2) the kneading method, which has the advantage of recovering almost all host and guest components (even in complexed or noncomplexed forms).

### Co-crystallization from ethanol-water mixture

Co-crystallization of the β-CD/ASO complex was performed in a 20 mL double-walled reactor, equipped with a magnetic stirring system, reflux condenser and dropping funnel. The host–guest molar ratio was 1:1 and 3:1 (considering that the main fish oil components are triglycerides, which theoretically requires three CD molecules for molecular encapsulation of one guest molecule). The molar ratios were calculated based on a mean molar mass for triglycerides from ASO of 899.5 g/mol (according to GC–MS analysis) and 1310 g/mol for β-CD hydrate (according to KFT analysis of water content of commercial β-CD). First, 1 mmol (or 3 mmol) of β-CD hydrate (1.313 ± 0.0007 g or 3.939 ± 0.0004 g) was suspended in 12 mL of distilled water and heated to 50 °C in the reactor. Subsequently, 4 mL of an ethanolic solution containing 1 mmol of ASO (0.902 ± 0.001 g) was added over 15 min to the β-CD solution under vigorous stirring. After the addition was completed, stirring was continued for another 30 min at the same temperature. The β-CD/ASO complex was crystallized by controlled cooling from 50 °C to 25 °C with a cooling rate of 8 °C·min^−1^ and the crystallization process was completed at 4 °C overnight. The obtained β-CD/ASO complex crystals were filtered, washed with 2 mL ethanol, and dried in a desiccator over molecular sieves (4 Å, Merck & Co., Inc.). The β-CD/ASO complex was stored at 4 °C in sealed containers. All co-crystallized complexes were obtained in duplicate.

### Kneading method

For the kneading method the same molar ratios of β-CD/ASO as described for the co-crystallization method were used. The main difference was related to the quantity of solvents. In a 50 mL ceramic mortar preheated to 50 °C, β-CD hydrate (1.312 ± 0.001 g and 3.938 ± 0.0007 g) was suspended in either 1 mL or 2 mL of water (for 1:1 or 3:1 molar ratio, respectively). To this suspension, 0.5 mL of an ethanololic solution containing 0.902 ± 0.002 g of ASO was added and the mixture was thoroughly milled for 15 min. Afterwards the mixture was cooled to room temperature, washed with ethanol (1 mL) to remove surface oil, and dried to constant mass in a desiccator over MS 4Å. The kneaded complexes were prepared in duplicates.

### GC–MS analysis of the raw and degraded ASO

The FA profile of nondegraded and degraded ASO was obtained by GC–MS analysis of the corresponding methyl esters (as well as acetals and ketals of FA degradation compounds). For derivatization, approximately 20 mg of ASO or the corresponding sample was dissolved in 3 mL of boron trifluoride–methanol solution in a 50 mL round-bottomed flask equipped with a reflux condenser, and the mixture was refluxed for 2 min. After cooling, hexane (2 mL) was added and the mixture was further refluxed for 2 min. After cooling, the organic layer was separated at the top of the flask by adding a sufficient amount of saturated sodium chloride solution. The upper layer was directly collected in a GC vial, dried (Na_2_SO_4_) and analyzed. For GC–MS analysis, a GC Hewlett Packard 6890 Series gas chromatograph coupled with a Hewlett Packard 5973 Mass Selective Detector was used. A Zebron 5-MS column (30 m length, 0.25 mm inner diameter, and 0.25 μm film thickness) and a temperature program from 50 °C to 300 °C with a heating rate of 6 °C·min^−1^ were used. The injector and detector temperatures were set at 300 °C. The carrier gas was helium, the injection volume was 2 μL and a solvent delay time of 7 min was set up for the GC. The EI energy of the MS system was 70 eV and the source temperature was 150 °C. A scan range of 50–300 amu and a scan rate of 1 s^−1^ were used. The identification of the main compounds from the raw and degraded ASO was performed by using three methods: the experimental MS spectra were compared with the NIST/EPA/NIH Mass Spectral Library 2.0 (2002), by using the FAMEs 37 standard mixture analyzed under the same conditions and by comparing the Kovats indices (obtained with the C_8_–C_20_ alkane standard) for the known FAMEs for this GC column type. The acquisition and handling of the GC–MS data was performed by using the Enhanced MSD ChemStation ver. D.02.00.275/2005 package (Agilent Technologies).

### TG analysis of β-CD/ASO complexes

The behavior of β-CD/ASO complexes during heating was evaluated by TG analysis. A Netzsch TG 209 apparatus, with a temperature program of 25–400 °C and a heating rate of 10.0 °C·min^−1^ were used. The analysis was performed under nitrogen. The acquisition and handling of the data from the thermogravimeter were performed by using Netzsch Proteus – Thermal Analysis version 6.1 software.

### DSC analysis of β-CD/ASO complexes

Calorimetric effects during heating of the β-CD/ASO complexes were evaluated by DSC analysis using a Netzsch DSC 204 apparatus. The temperature program was 25–400 °C, with a heating rate of 10.0 °C·min^−1^, a purge flow of 20 mL·min^−1^, and a protective flow of 50 mL·min^−1^. All DSC determinations were performed under nitrogen atmosphere.

### KFT analysis of β-CD/ASO complexes

The water content of β-CD/ASO complexes was determined by the bi-component technique of volumetric KFT. A KF 701 Titrino apparatus, equipped with a 10 mL dosing system and coupled with a 703 Ti Stand stirring system (both from Metrohm, Herisau, Switzerland) was used. A complex of up to 100 mg was used for KFT analysis. The bulk solvent volume (component 2, Hydranal-Solvent, Sigma-Aldrich) was 30 mL at the start of the analysis. Hydranal-Titrant 5 was used as component 1 (Sigma-Aldrich). The titer of the component 1 was determined by using Hydranal-Water Standard 10.0 mg H_2_O·g^−1^ (Sigma-Aldrich). An electrode polarization of 50 μA, endpoint voltage of 250 mV, maximum titration rate of 5 mL·min^−1^, drift as stop criterion, a drift value of 20 μL·min^−1^, and an extraction time of 300 s were the values for the main KFT parameters.

### Statistical and regression analyses

Statistical evaluation of the data from GC–MS, TG, DSC, and KFT analyses was performed by means of the ANOVA approach. Regression analysis was used for TG, DSC–KFT dependence. For the regression equation, the Pearson correlational coefficient, *r*^2^, *F*-test, and standard errors for both equations and regression coefficients were determined.

## Supporting Information

File 1 GC–MS analysis of all raw and degraded ASO, as well as TG, DSC and KFT data for the β-CD/ASO complexes.
