# Supplementary material for: Thermal and oxidative stability of Atlantic salmon oil (Salmo salar L.) and complexation with β-cyclodextrin
Source: Beilstein J Org Chem. 2016 Feb 2;12:179–91. doi: 10.3762/bjoc.12.20 (PMC4778528; doi:10.3762/bjoc.12.20)
Supplement: File 1 — GC–MS analysis of all raw and degraded ASO, as well as TG, DSC and KFT data for the β-CD/ASO complexes. [file Beilstein_J_Org_Chem-12-179-s001.pdf]

Supporting Information

for

Thermal and oxidative stability of Atlantic salmon oil (*Salmo salar* L.) and complexation with β-cyclodextrin

Daniel I. Hădărugă[1,§*], Mustafa Ünlüsayin[2], Alexandra T. Gruia[3], Cristina Birău (Mitroi)[4], Gerlinde Rusu[1], Nicoleta G. Hădărugă[4]

Address: [1]Department of Applied Chemistry, Organic and Natural Compounds Engineering, Polytechnic University of Timişoara, Carol Telbisz 6, 300001-Timişoara, Romania, [2]Department of Fish Processing Technology, Akdeniz University, Dumlupinar Boulevard, Campus Antalya, 07058-Antalya, Turkey, [3]Regional Centre for Immunology and Transplant, County Clinical Emergency Hospital Timişoara, Iosif Bulbuca Blvd. 10, 300736-Timişoara, Romania and [4]Department of Food Science, Banat's University of Agricultural Sciences and Veterinary Medicine "King Mihai I of Romania" - Timişoara, Calea Aradului 119, 300645-Timişoara, Romania

[§]Phone +40-256-404224; Fax: +40-256-403060

Email: Daniel I. Hădărugă - daniel.hadaruga@upt.ro

[*]Corresponding author

GC–MS analysis of all raw and degraded ASO, as well as TG, DSC and KFT data for the β-CD/ASO complexes

GC–MS analysis

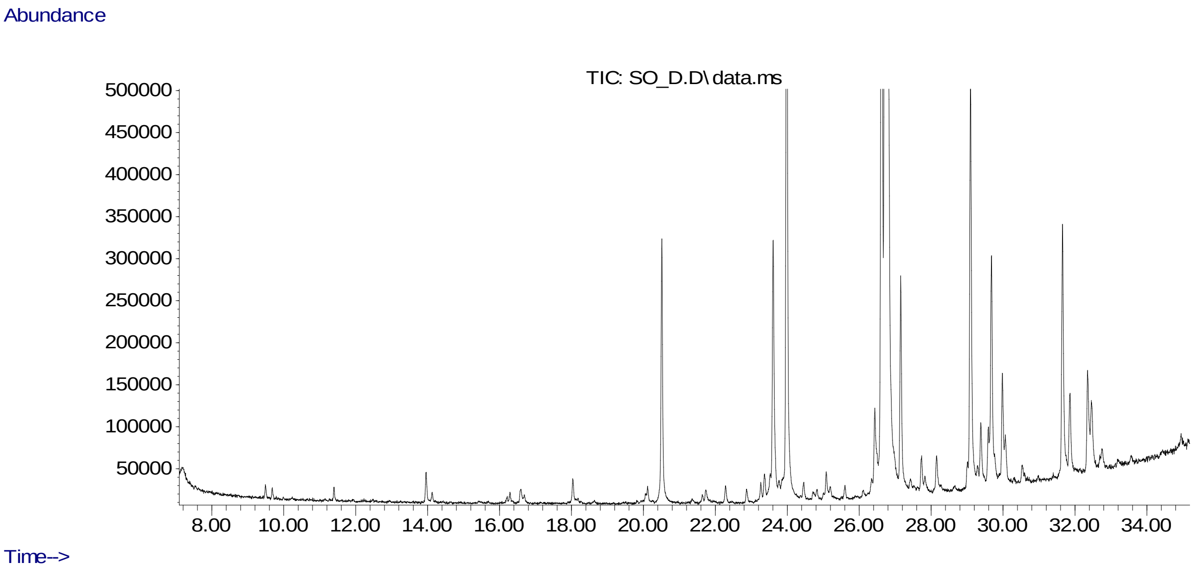

Figure S1: GC chromatogram from the GC–MS analysis of the raw Atlantic salmon oil (*ASOa*).

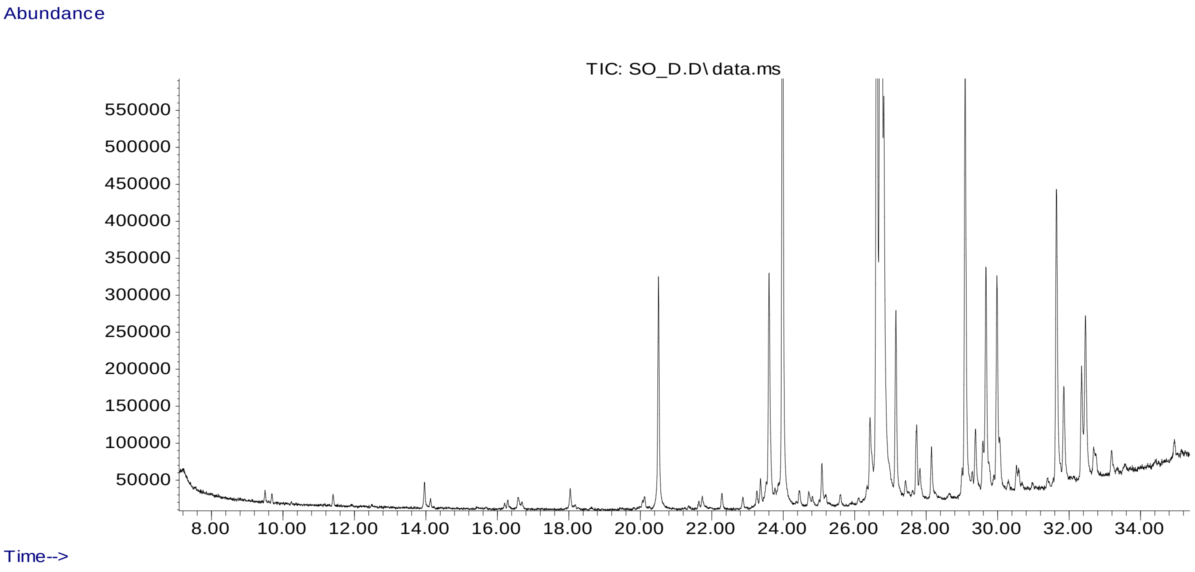

Figure S2: GC chromatogram from the GC–MS analysis of the raw Atlantic salmon oil duplicate (*ASOb*).

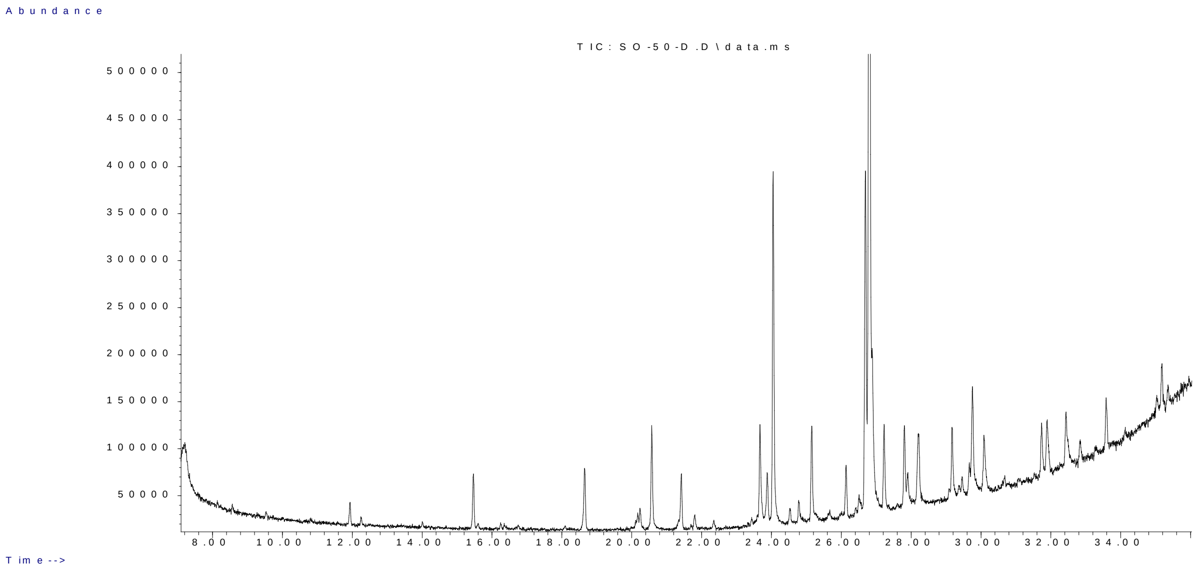

Figure S3: GC chromatogram from the GC–MS analysis of the Atlantic salmon oil degraded at low temperature (50 °C) (*ASO50a*).

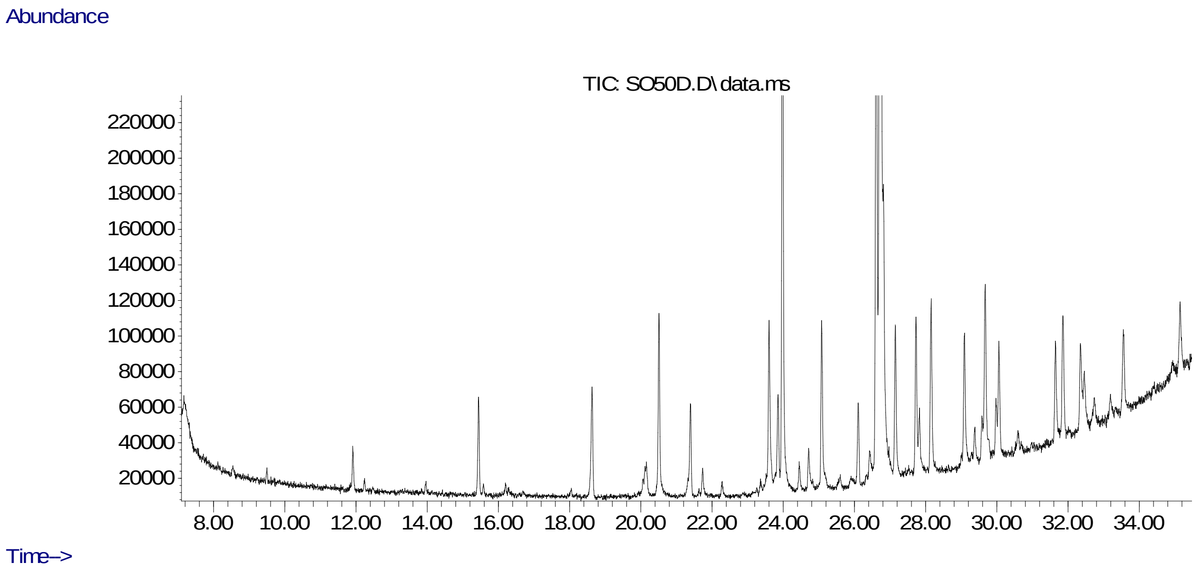

Figure S4: GC chromatogram from the GC–MS analysis of the Atlantic salmon oil degraded at low temperature, duplicate (50 °C) (*ASO50b*).

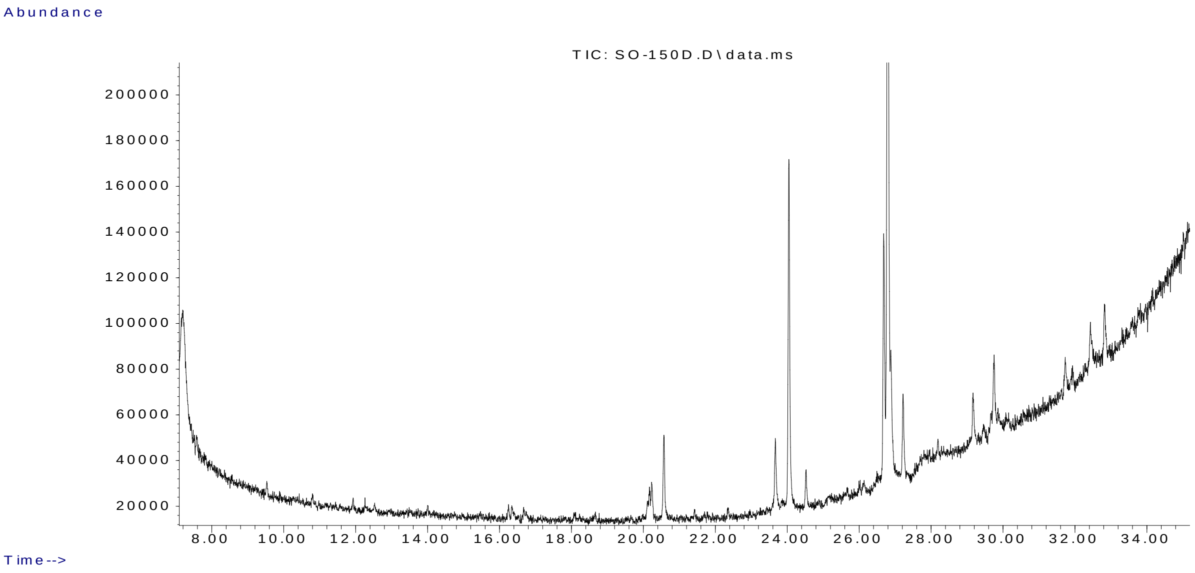

Figure S5: GC chromatogram from the GC–MS analysis of the Atlantic salmon oil degraded at high temperature (150 °C) (*ASO150a*).

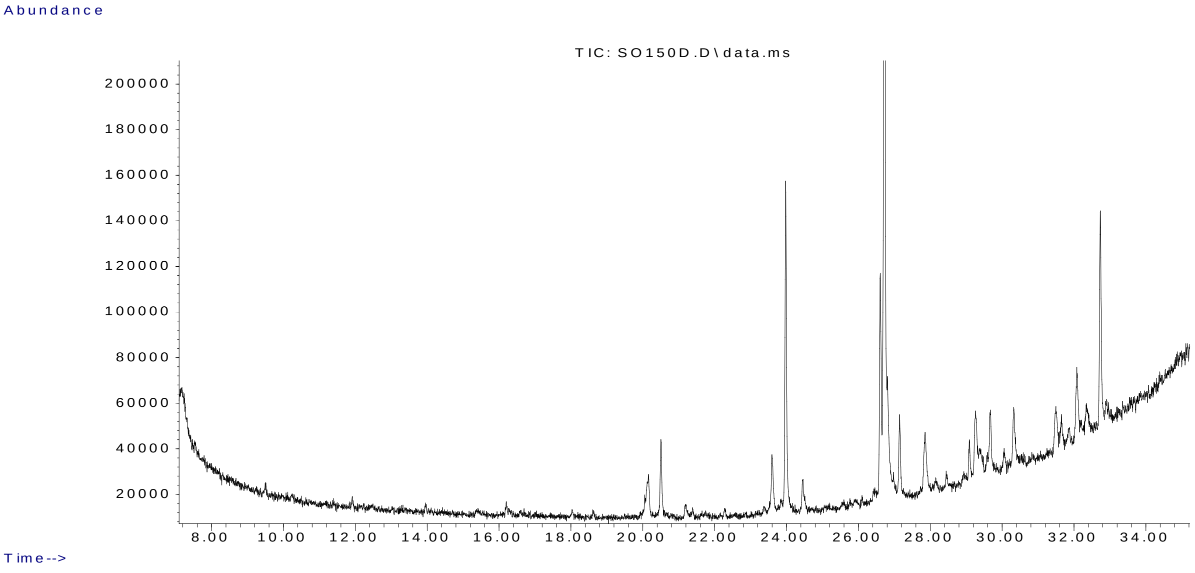

Figure S6: GC chromatogram from the GC–MS analysis of the Atlantic salmon oil degraded at high temperature, duplicate (150 °C) (*ASO150b*).

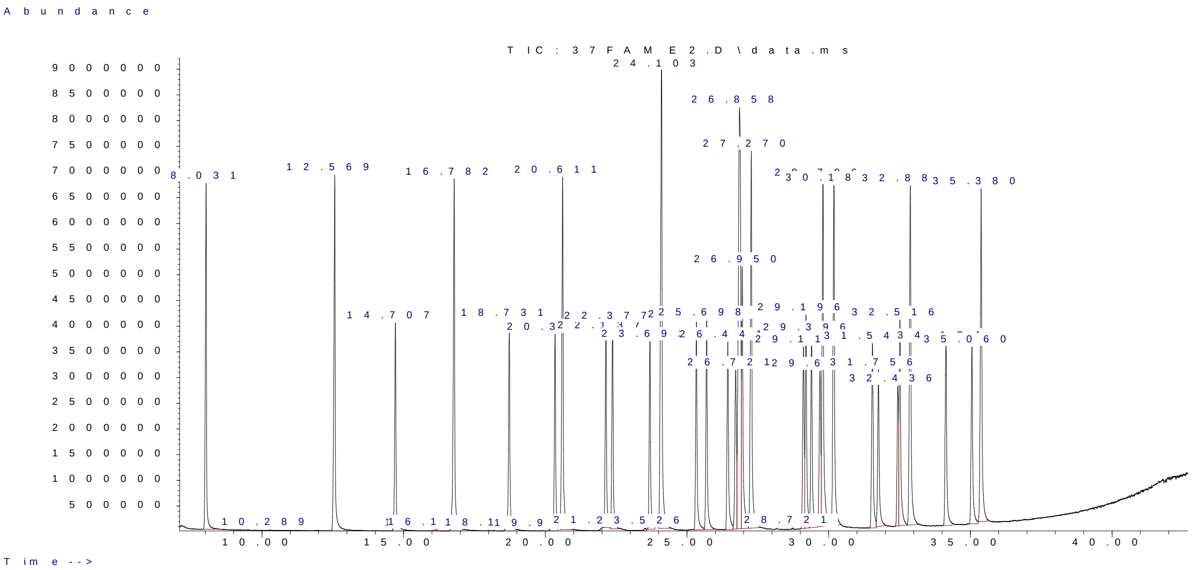

Figure S7: GC chromatogram from the GC–MS analysis of the fatty acid methyl esters (FAMEs) standard solution.

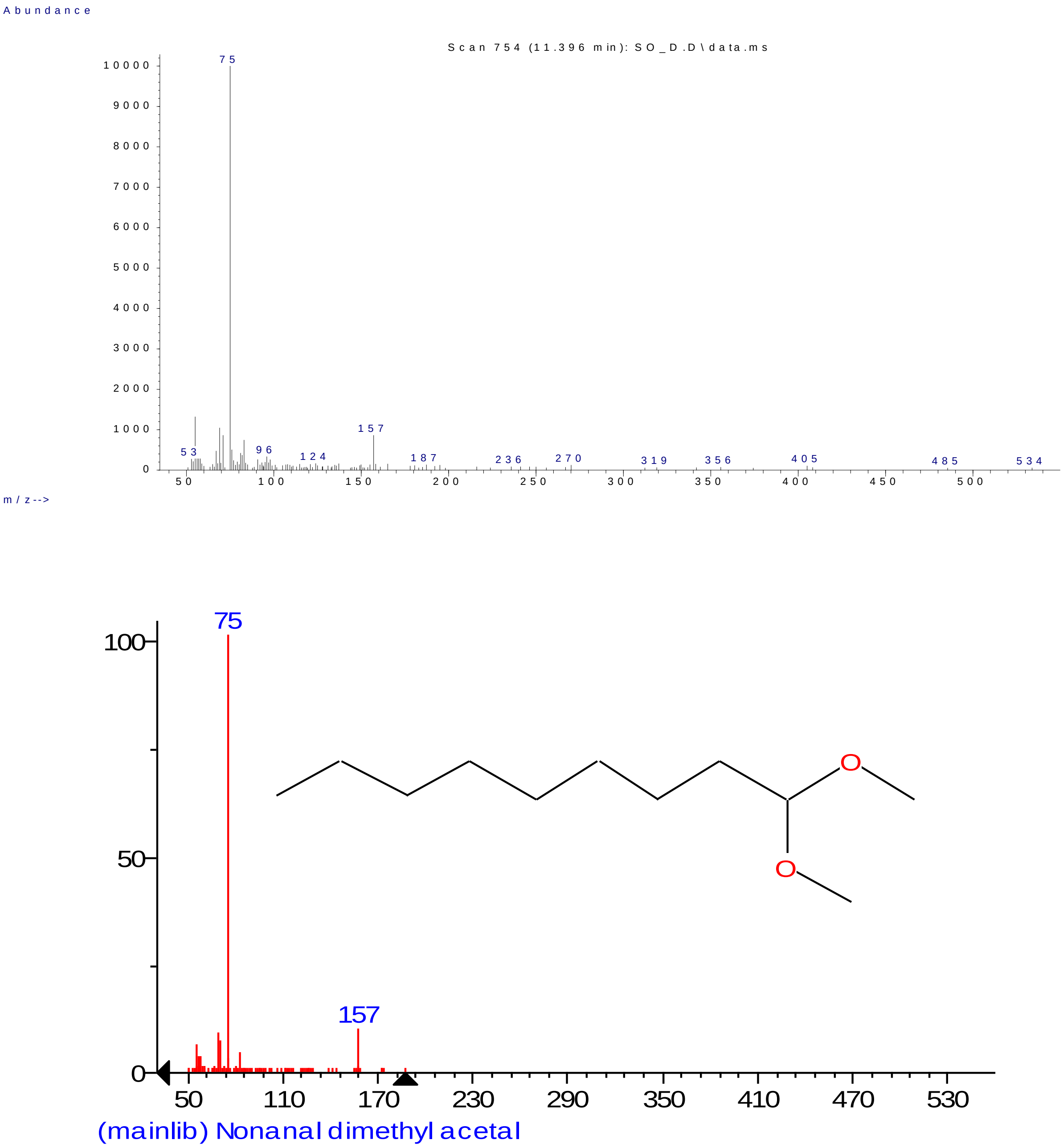

Figure S8: Experimental (up) and from the NIST database (down) MS spectra for nonanal (dimethyl acetal).

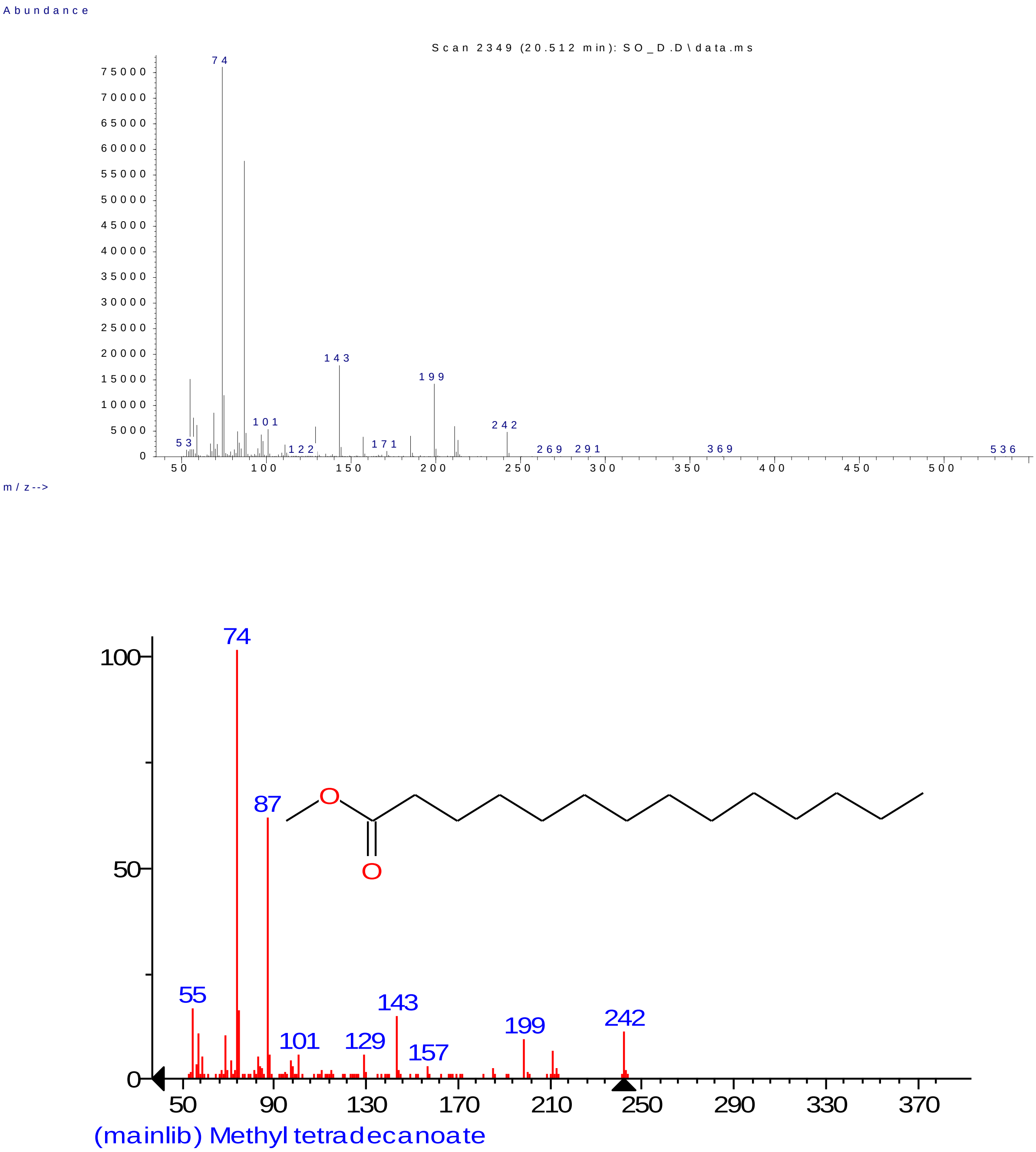

Figure S9: Experimental (up) and from the NIST database (down) MS spectra for myristic acid methyl ester.

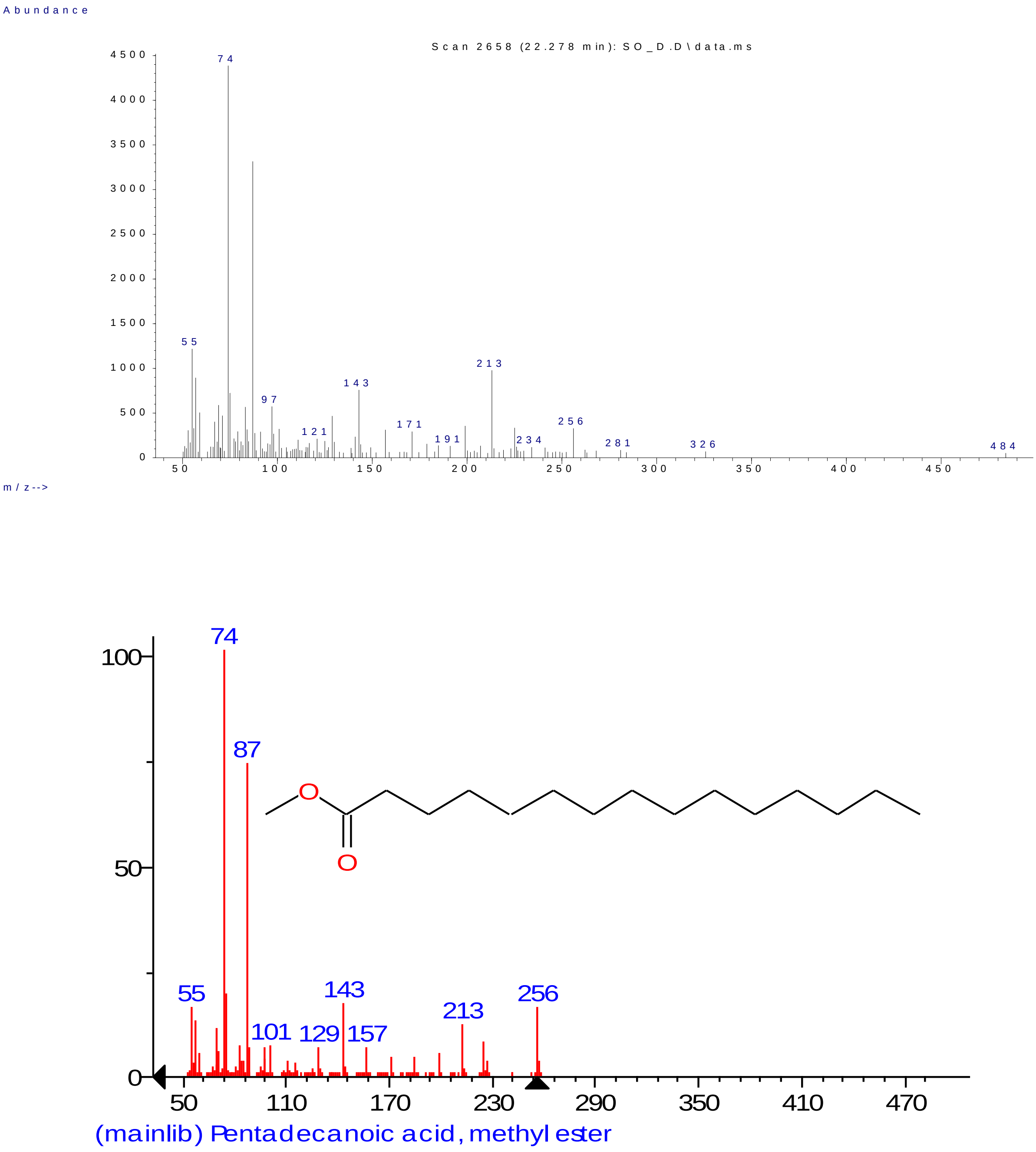

Figure S10: Experimental (up) and from the NIST database (down) MS spectra for pentadecanoic acid methyl ester.

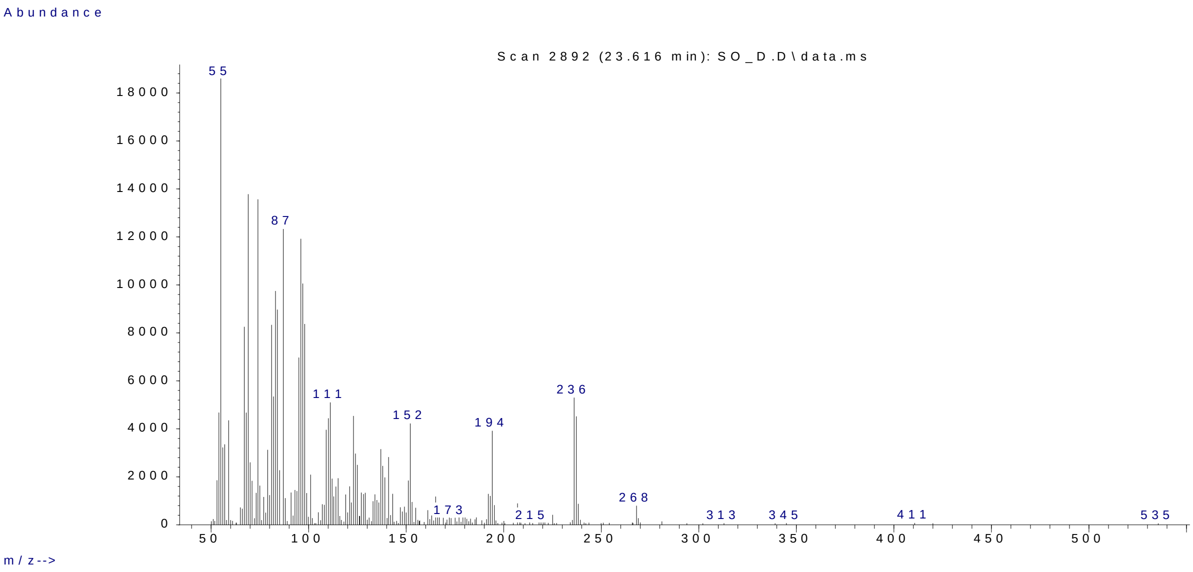

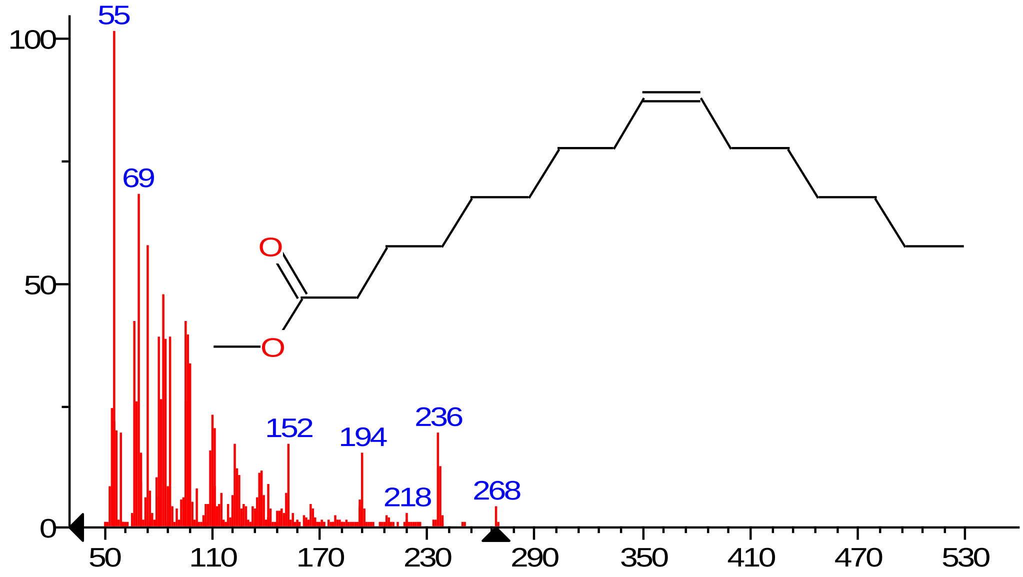

Figure S11: Experimental (up) and from the NIST database (down) MS spectra for palmitoleic acid methyl ester.

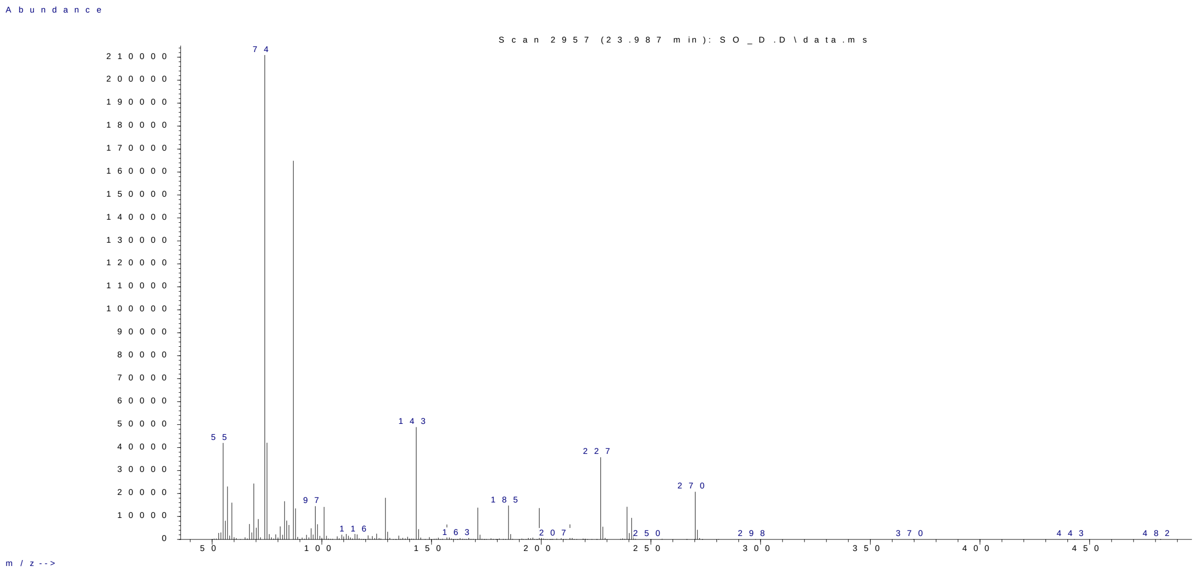

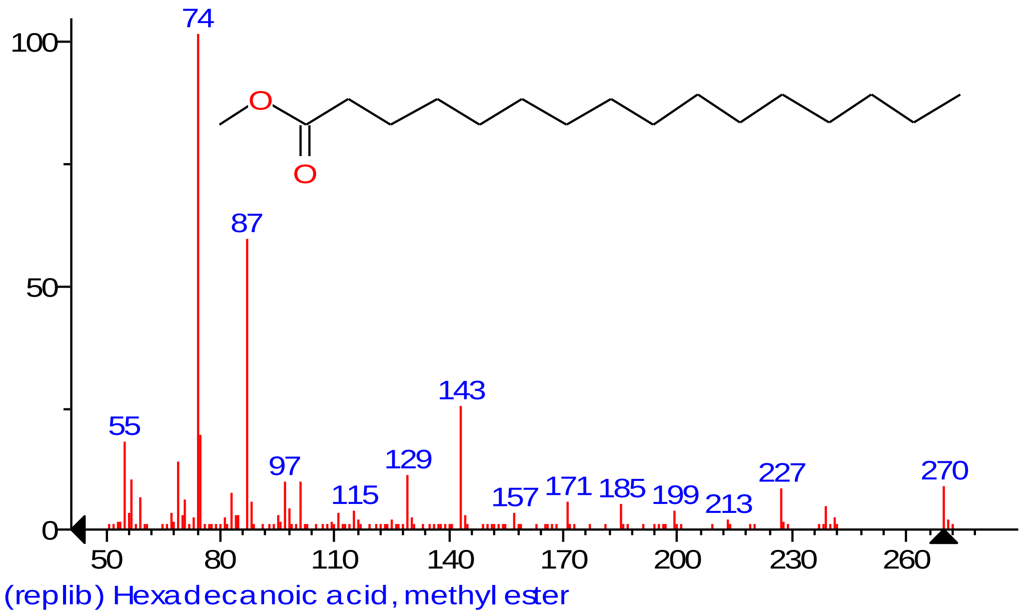

Figure S12: Experimental (up) and from the NIST database (down) MS spectra for palmitic acid methyl ester.

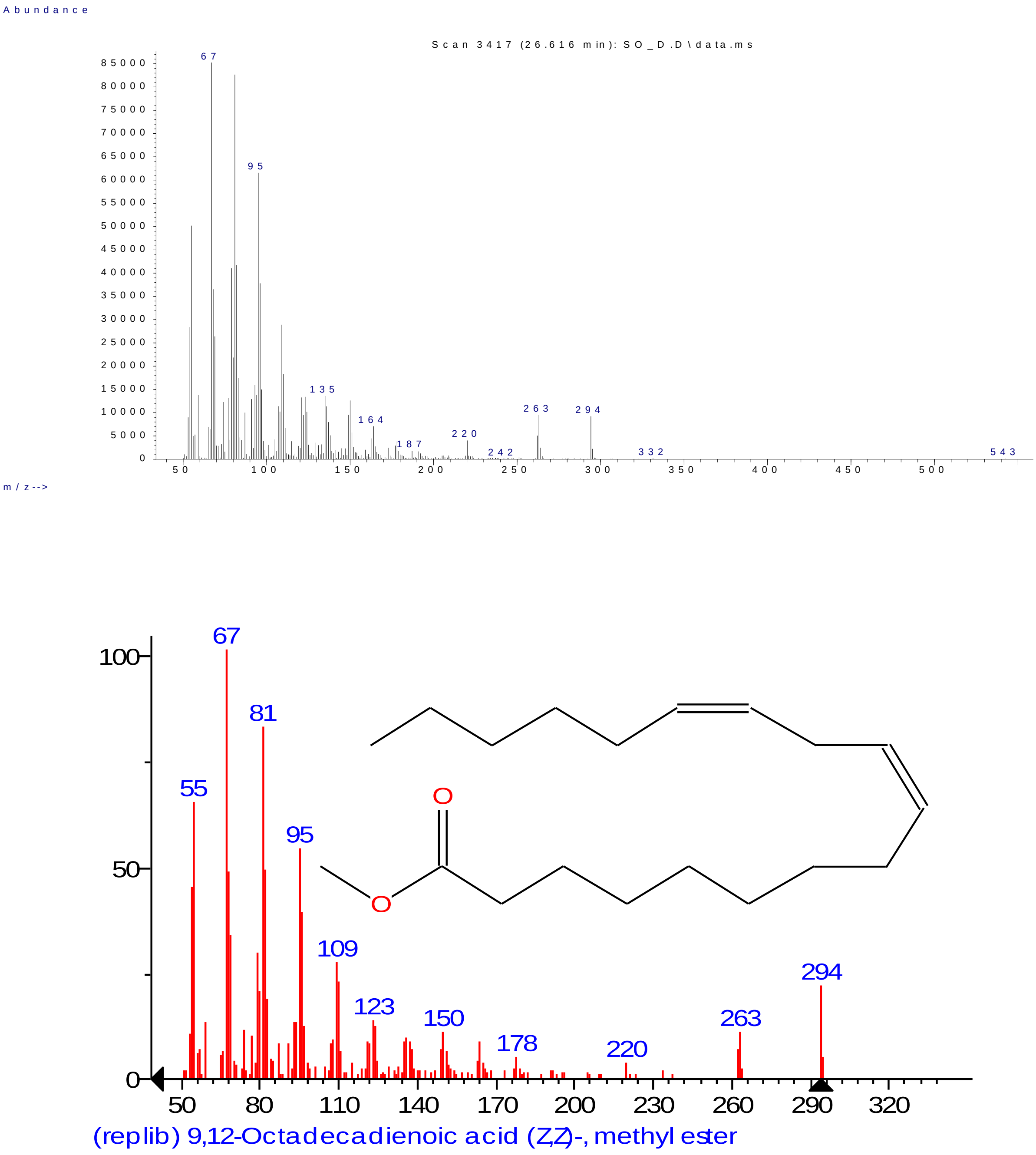

Figure S13: Experimental (up) and from the NIST database (down) MS spectra for linoleic acid methyl ester.

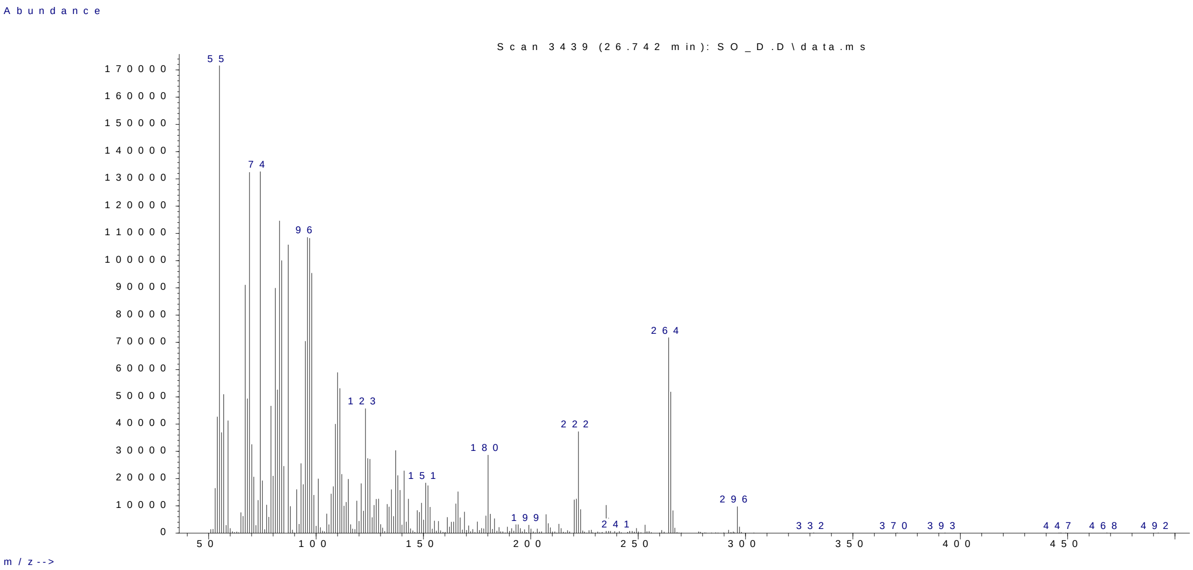

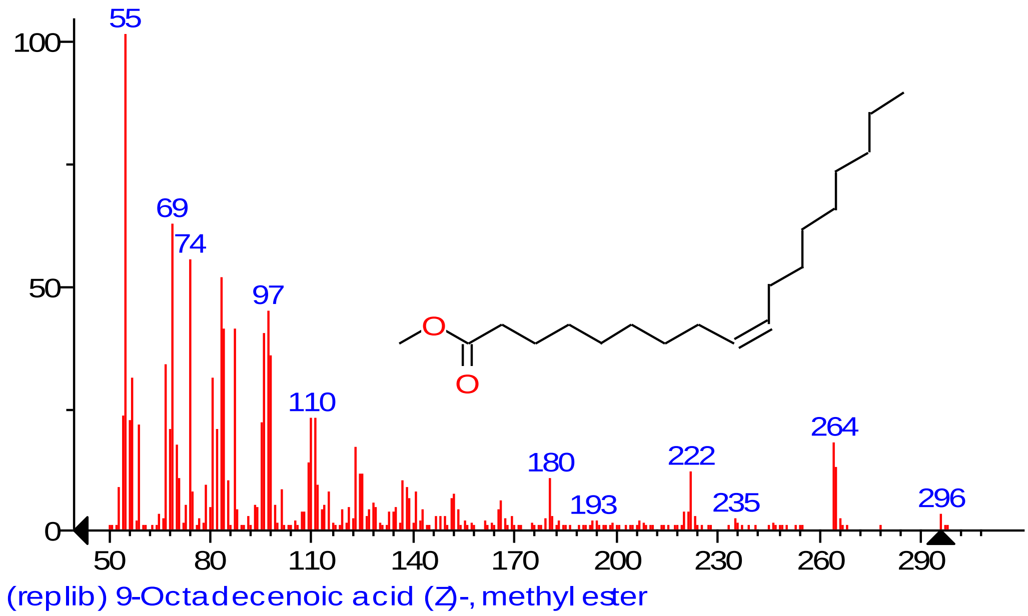

Figure S14: Experimental (up) and from the NIST database (down) MS spectra for oleic acid methyl ester.

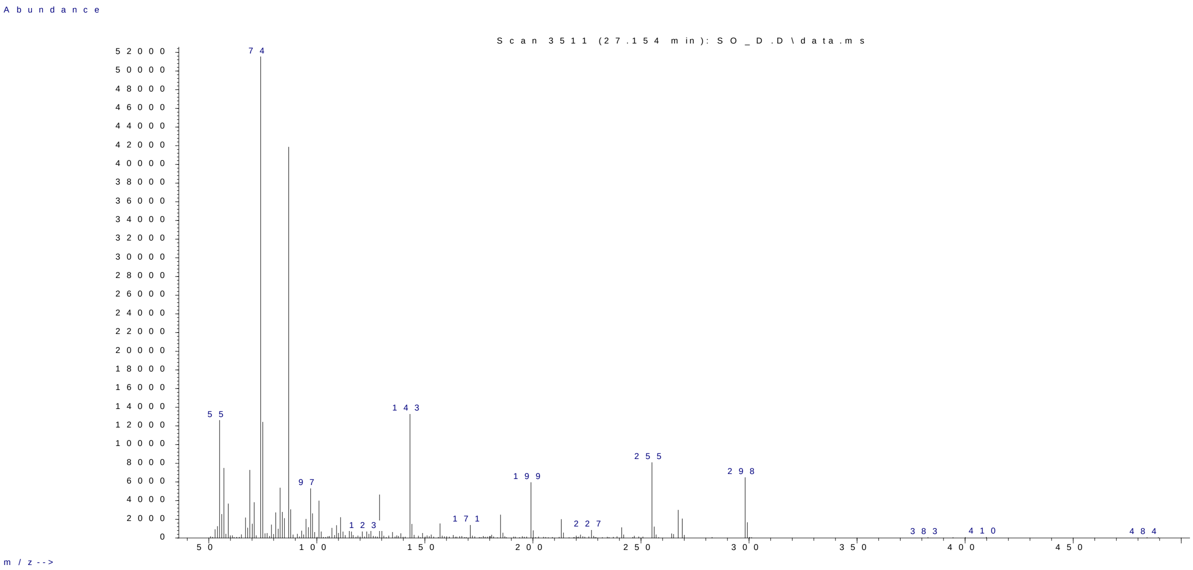

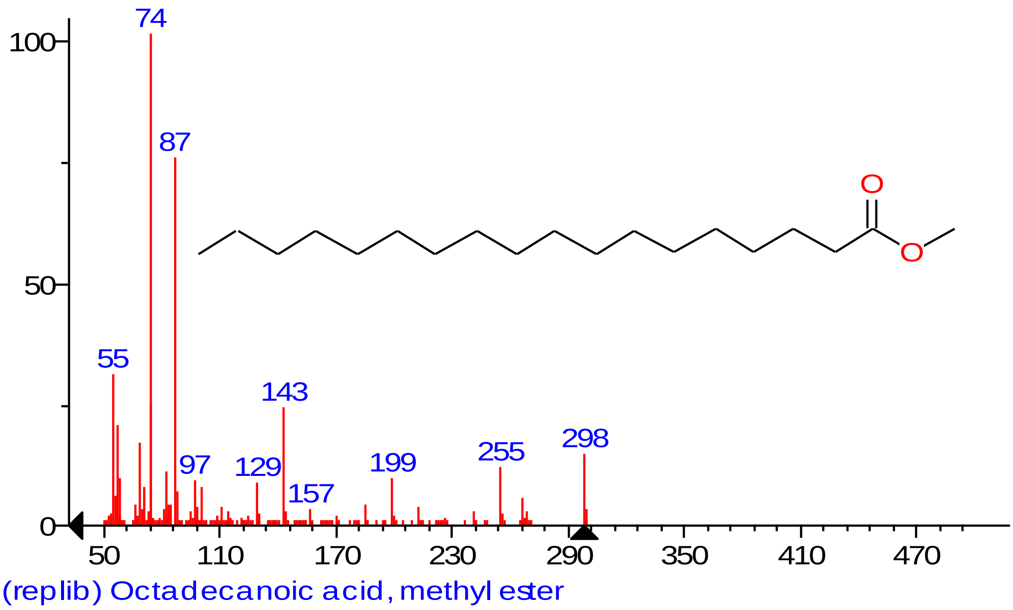

Figure S15: Experimental (up) and from the NIST database (down) MS spectra for stearic acid methyl ester.

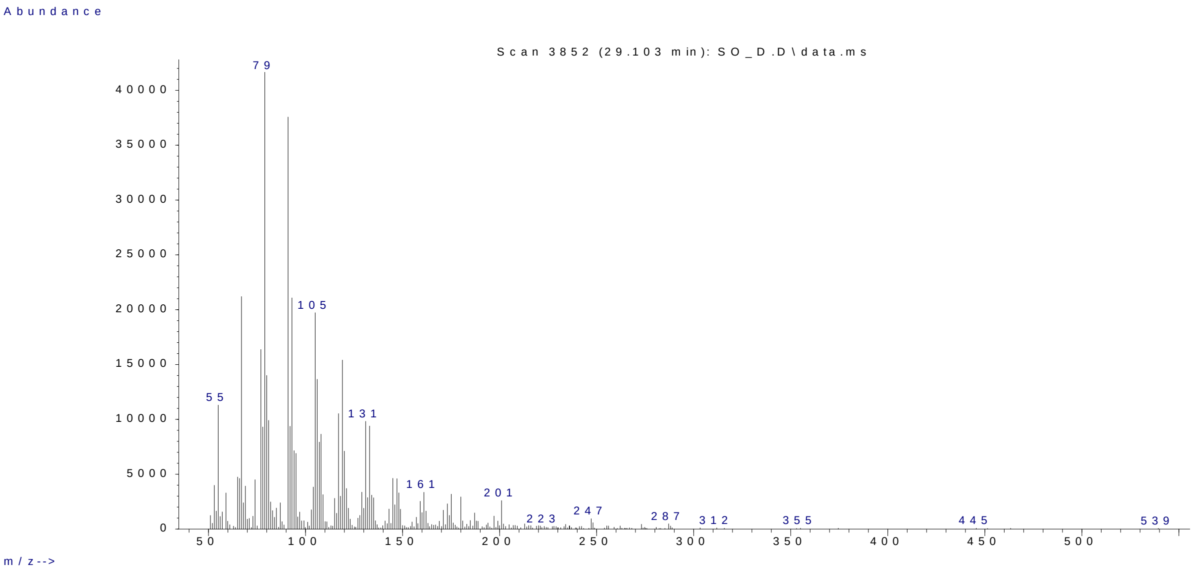

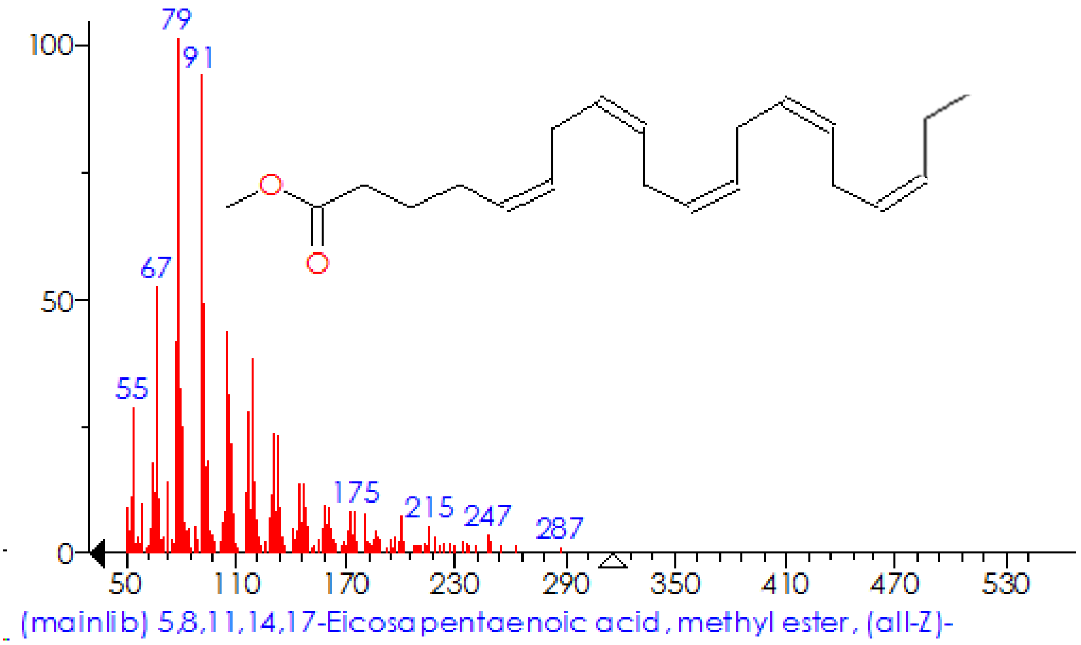

Figure S16: Experimental (up) and from the NIST database (down) MS spectra for (all-*Z*)-5,8,11,14,17-eicosapentaenoic acid methyl ester (EPA methyl ester).

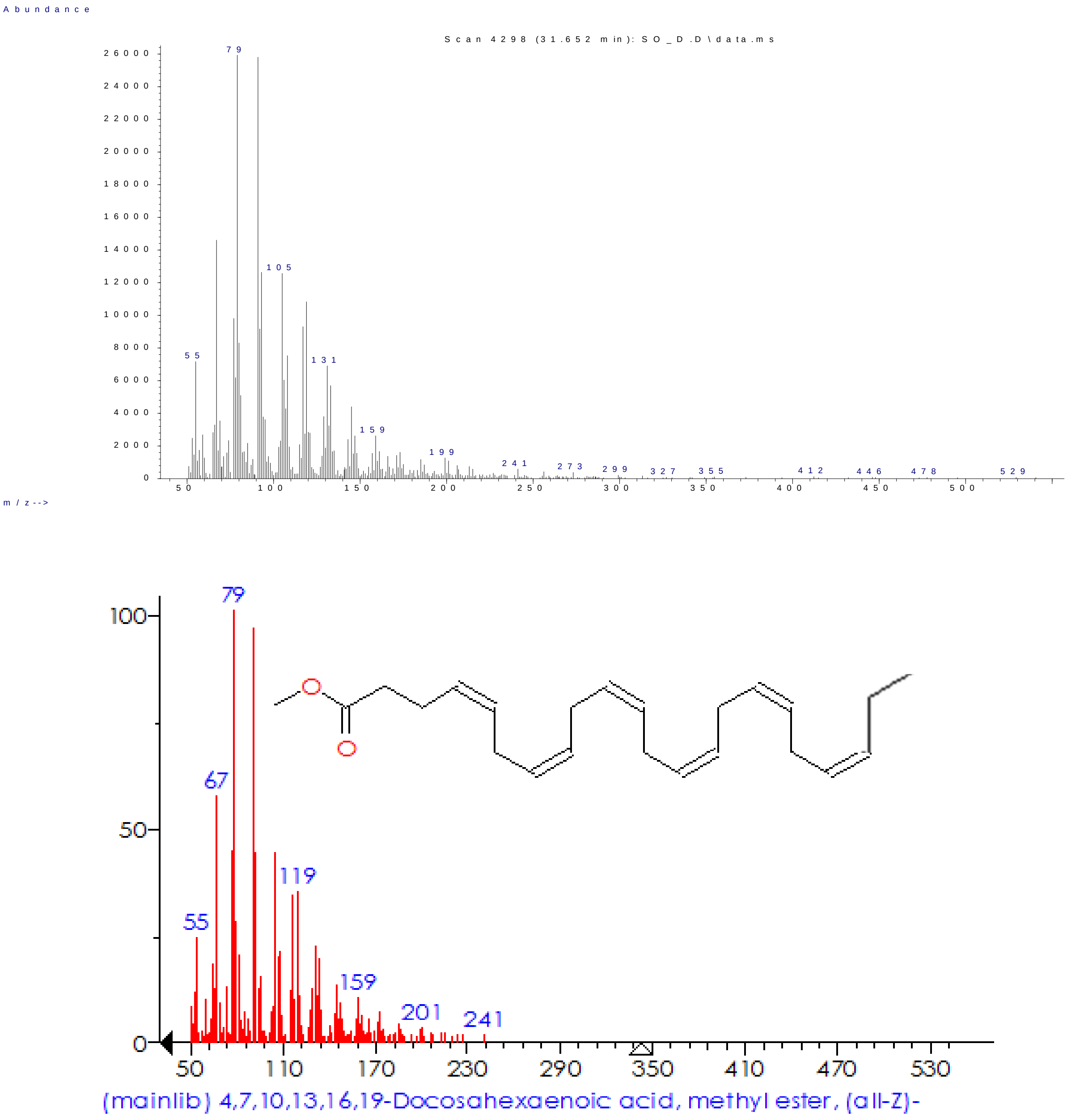

Figure S17: Experimental (up) and from the NIST database (down) MS spectra for (all-*Z*)-4,7,10,13,16,19-docosahexaenoic acid methyl ester (DHA methyl ester).

Thermogravimetric analysis

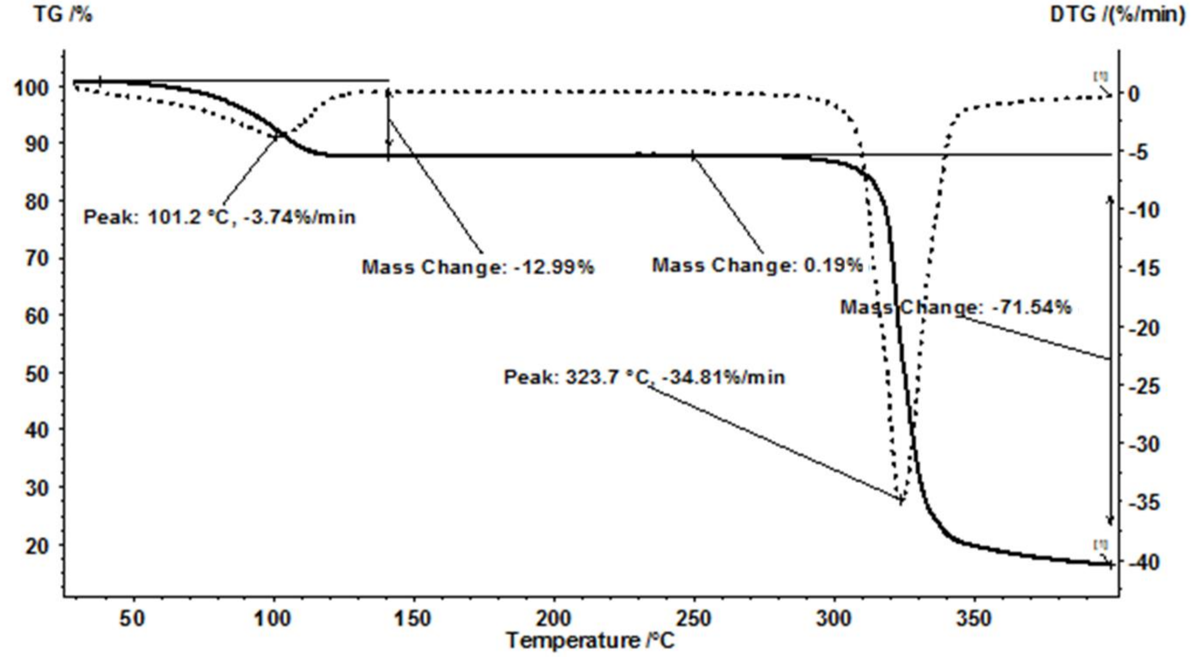

Figure S18: Thermogram from TG/DTG analysis of commercial β-cyclodextrin (*βCD*).

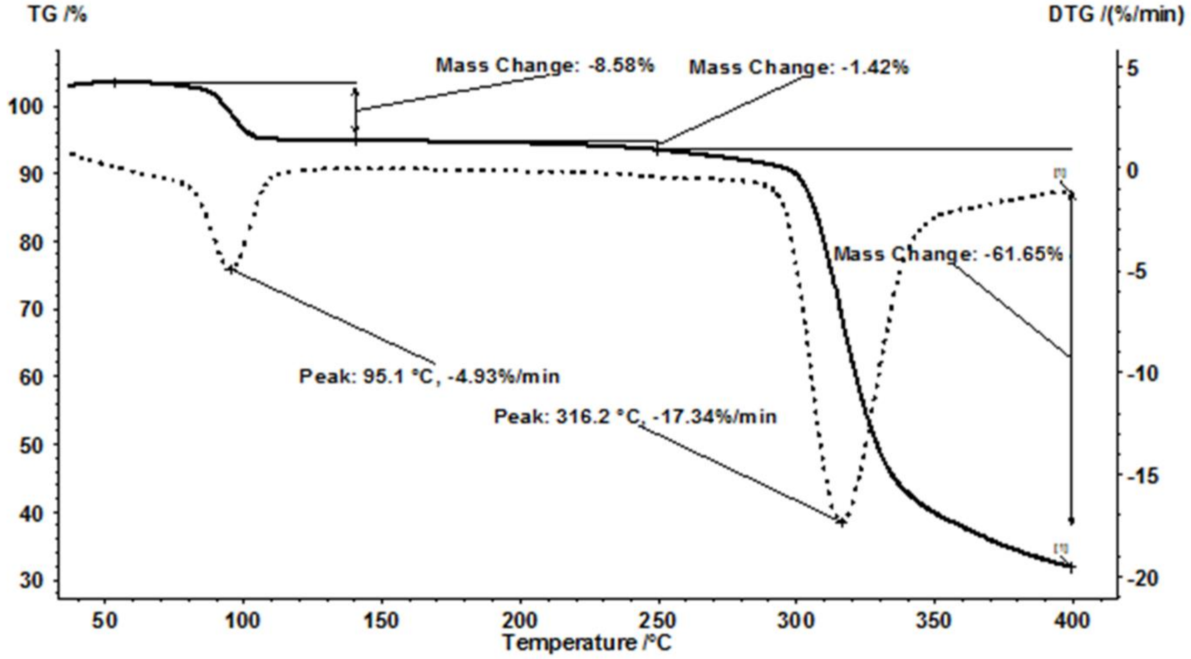

Figure S19: Thermogram from TG/DTG analysis of *β-CD/ASO_1:1_a* complex.

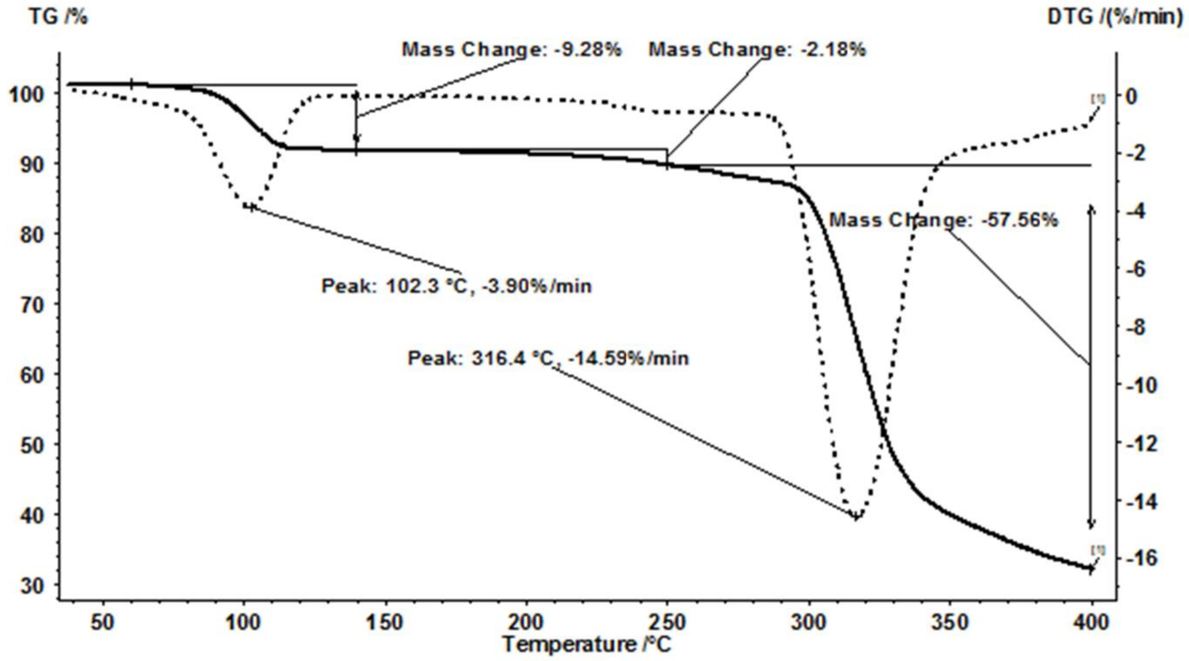

Figure S20: Thermogram from TG/DTG analysis of *β-CD/ASO_1:1_b* complex

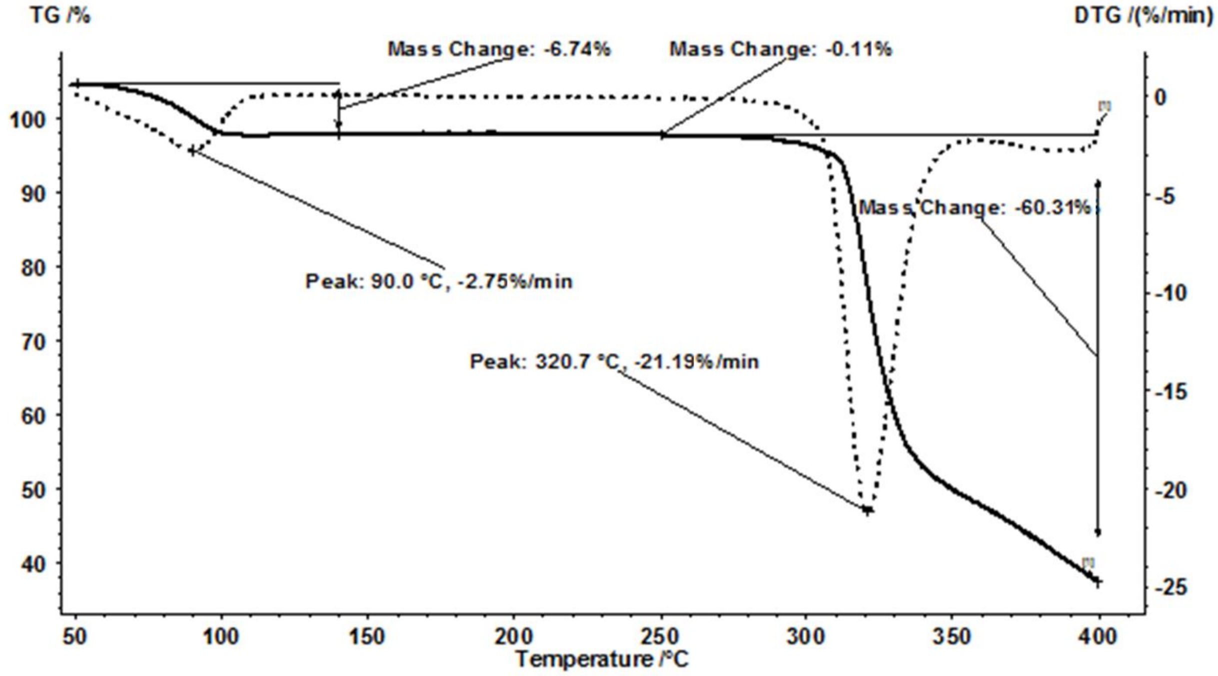

Figure S21: Thermogram from TG/DTG analysis of *β-CD/ASO_3:1_a* complex.

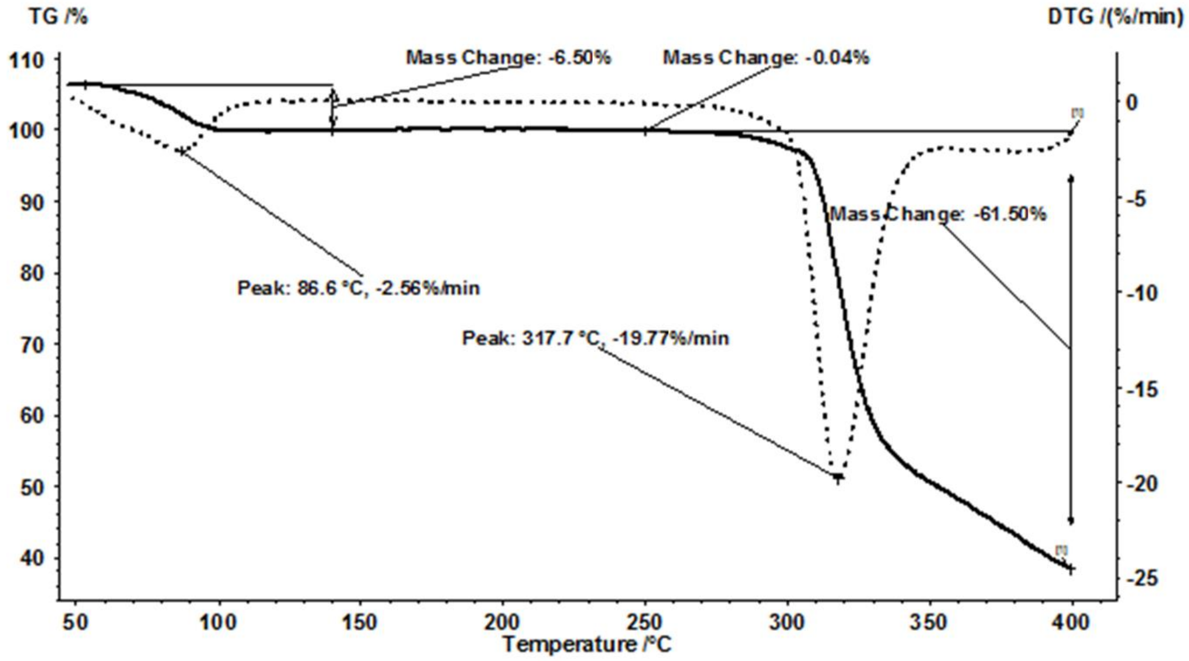

Figure S22: Thermogram from TG/DTG analysis of *β-CD/ASO_3:1_b* complex.

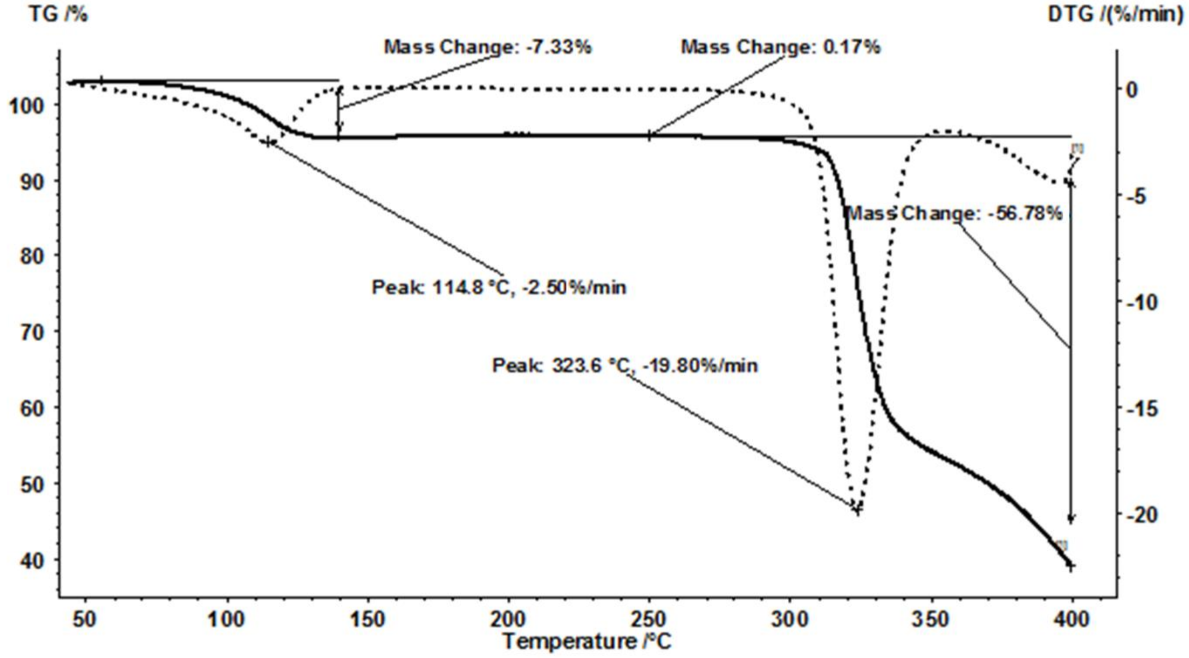

Figure S23: Thermogram from TG/DTG analysis of *β-CD/ASO_1:1(k)_a* complex.

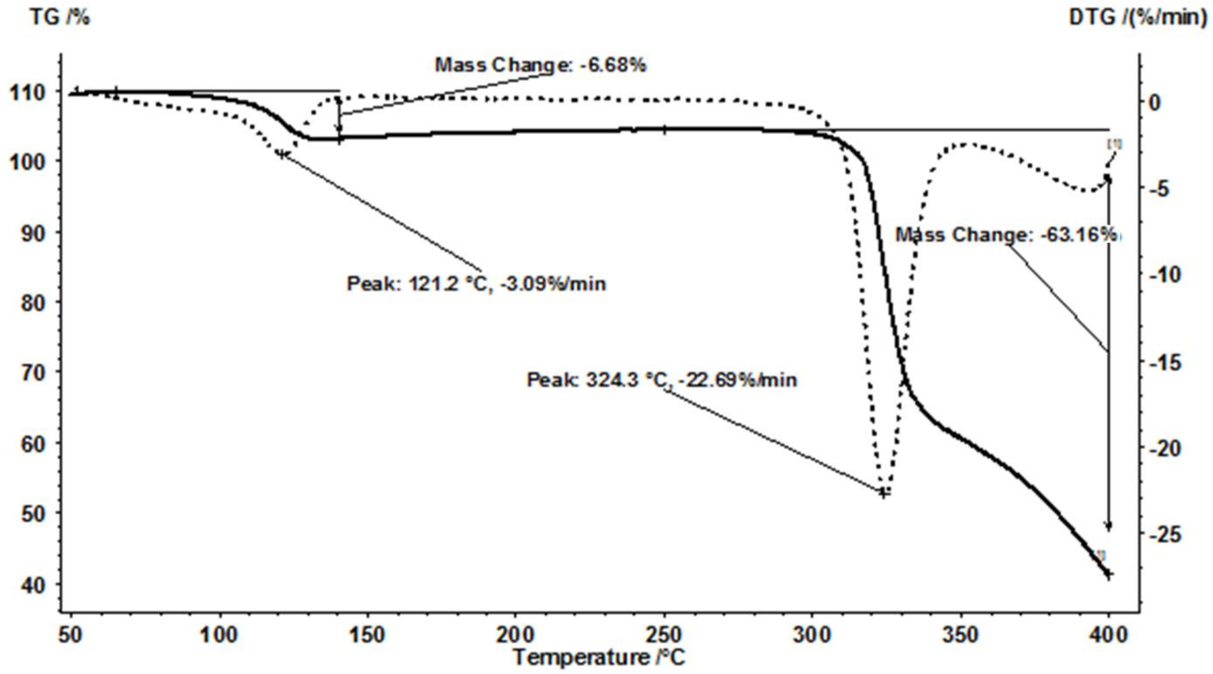

Figure S24: Thermogram from TG/DTG analysis of *β-CD/ASO_1:1(k)_b* complex.

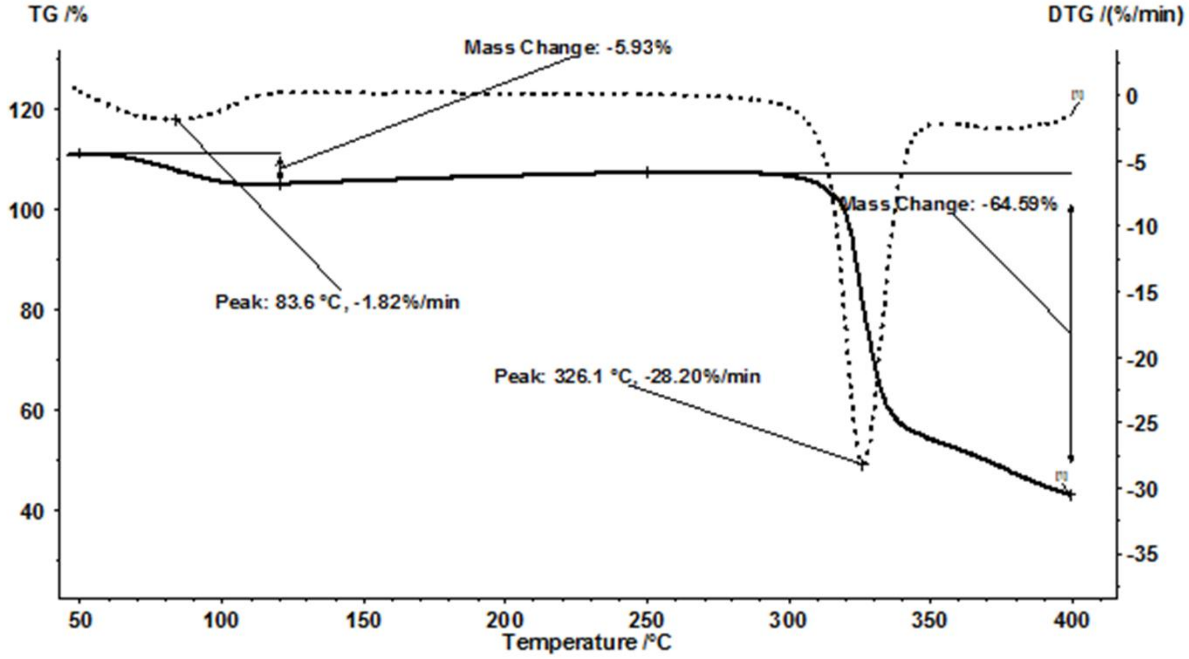

Figure S25: Thermogram from TG/DTG analysis of *β-CD/ASO_3:1(k)_a* complex.

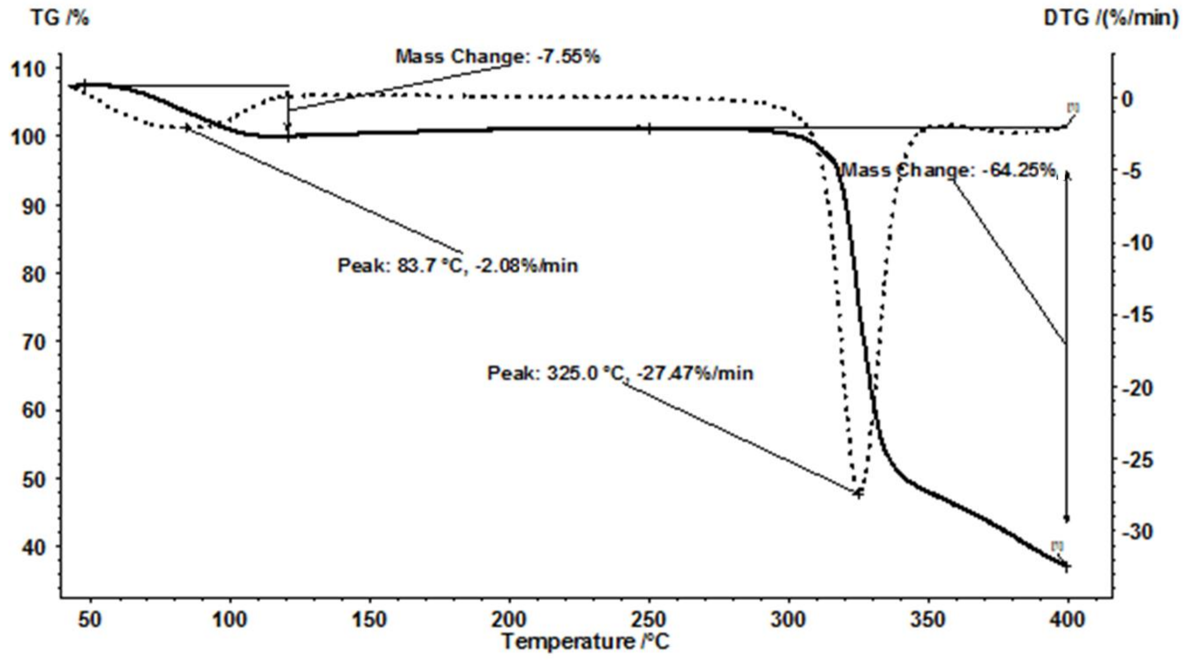

Figure S26: Thermogram from TG/DTG analysis of *β-CD/ASO_3:1(k)_b* complex.

Differential scanning calorimetry analysis

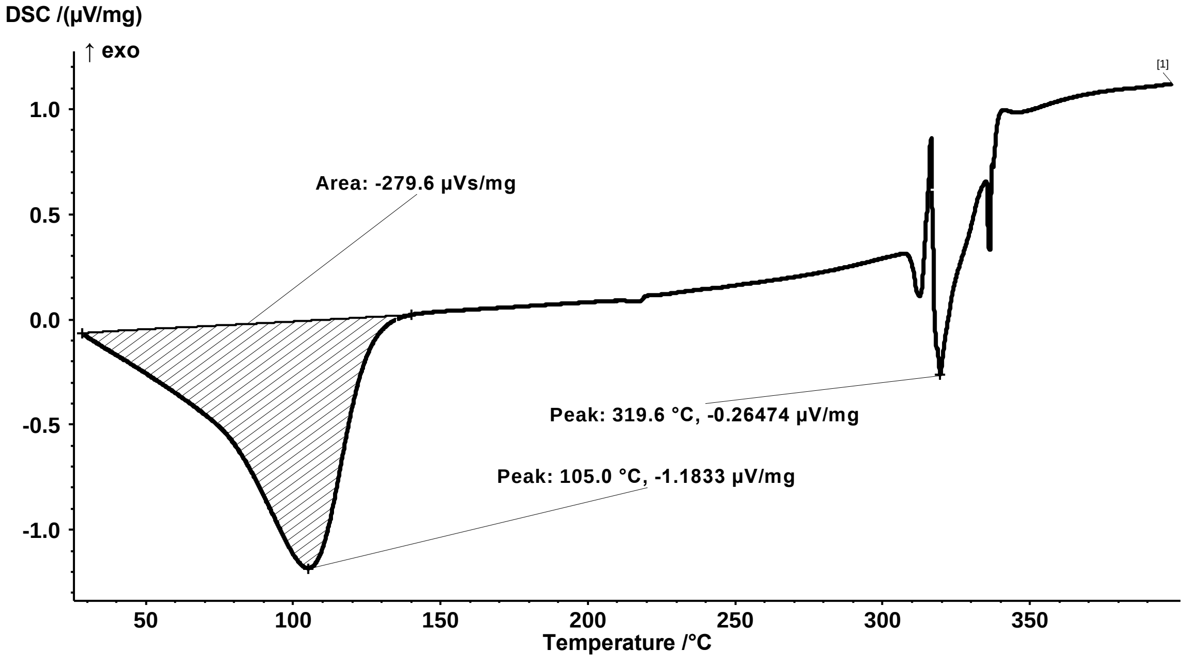

Figure S27: DSC analysis of commercial β-cyclodextrin (*β-CD*).

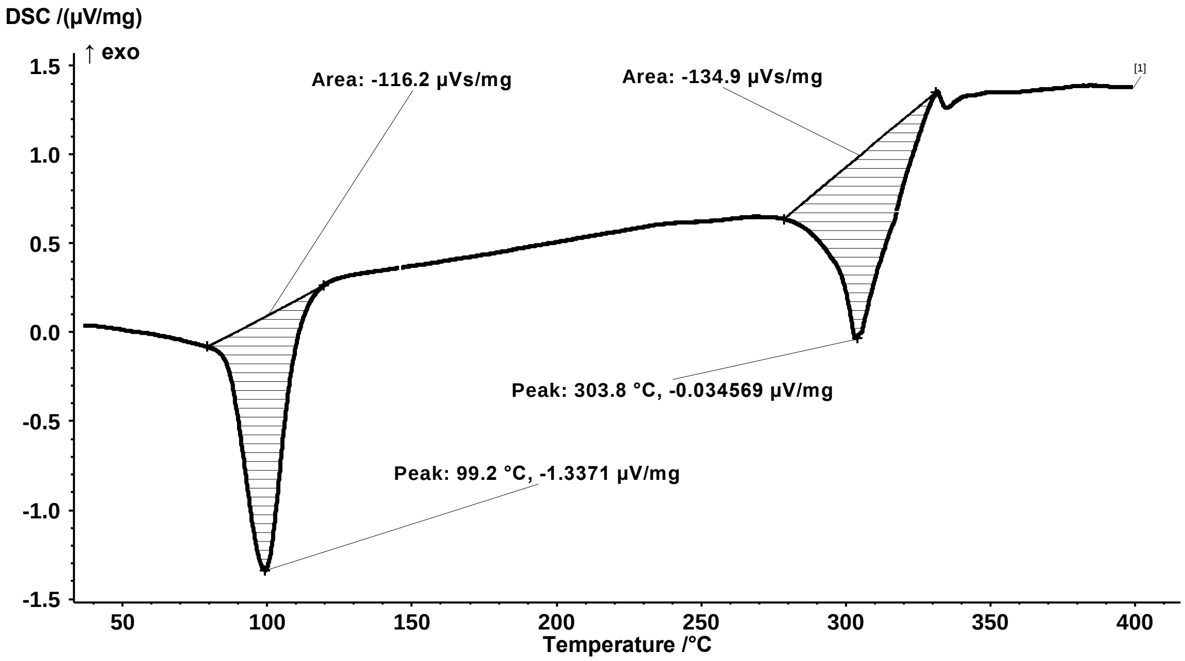

Figure S28: DSC analysis of *β-CD/ASO_1:1_a* complex.

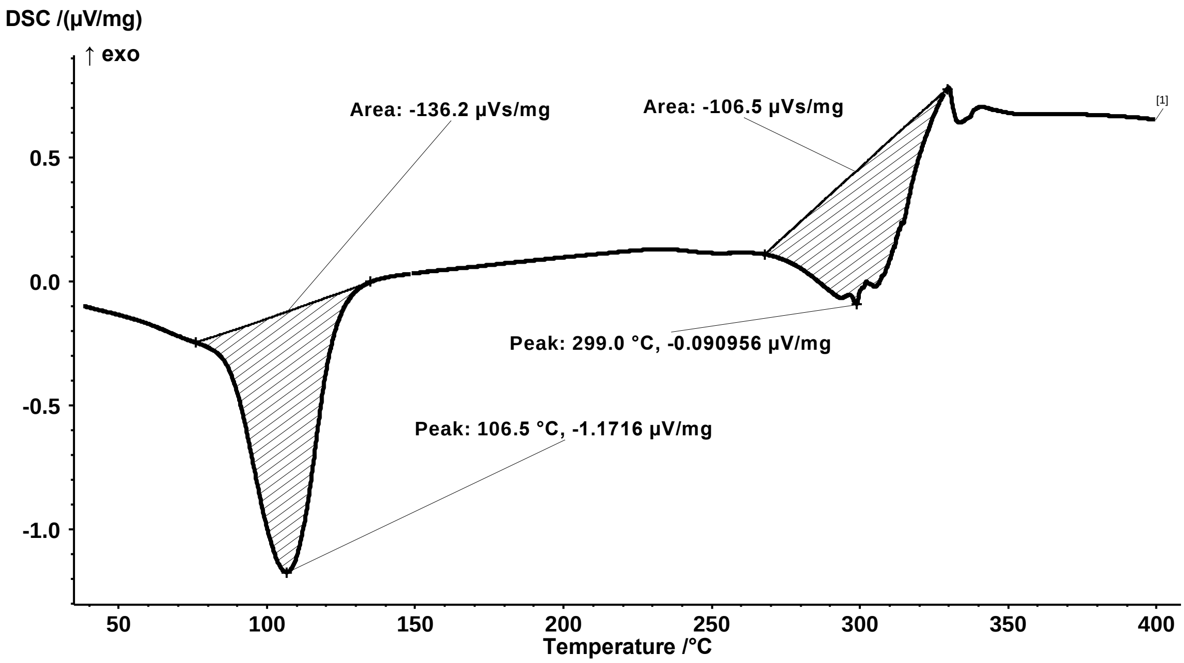

Figure S29: DSC analysis of *β-CD/ASO_1:1_b* complex.

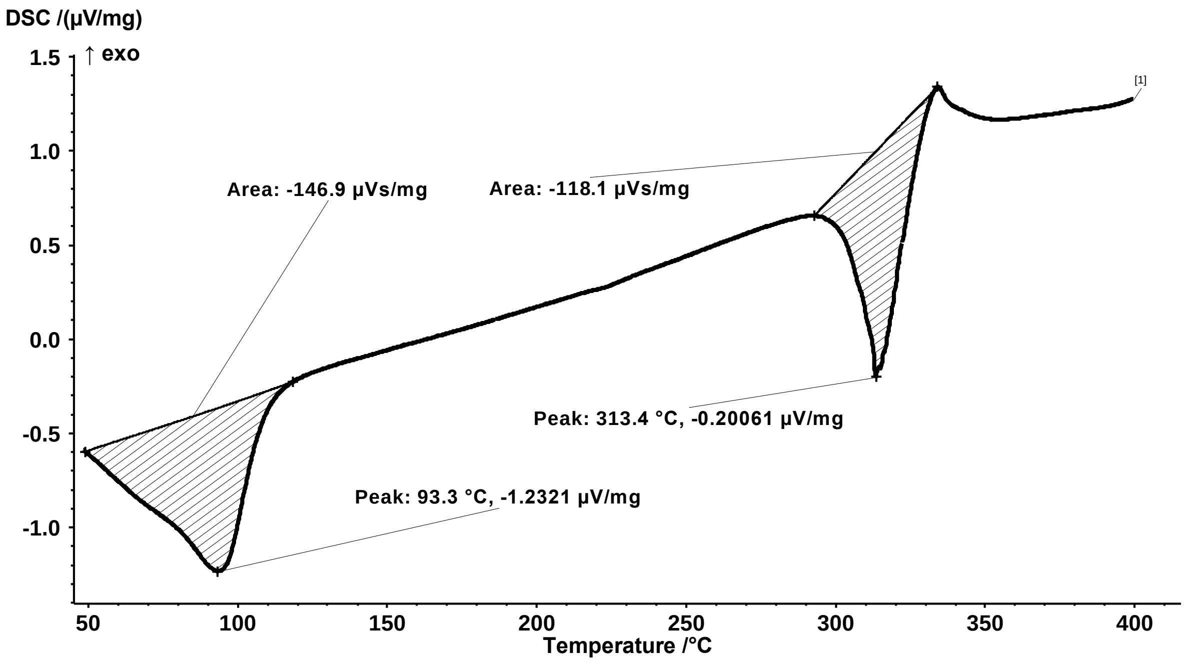

Figure S30: DSC analysis of *β-CD/ASO_3:1_a* complex.

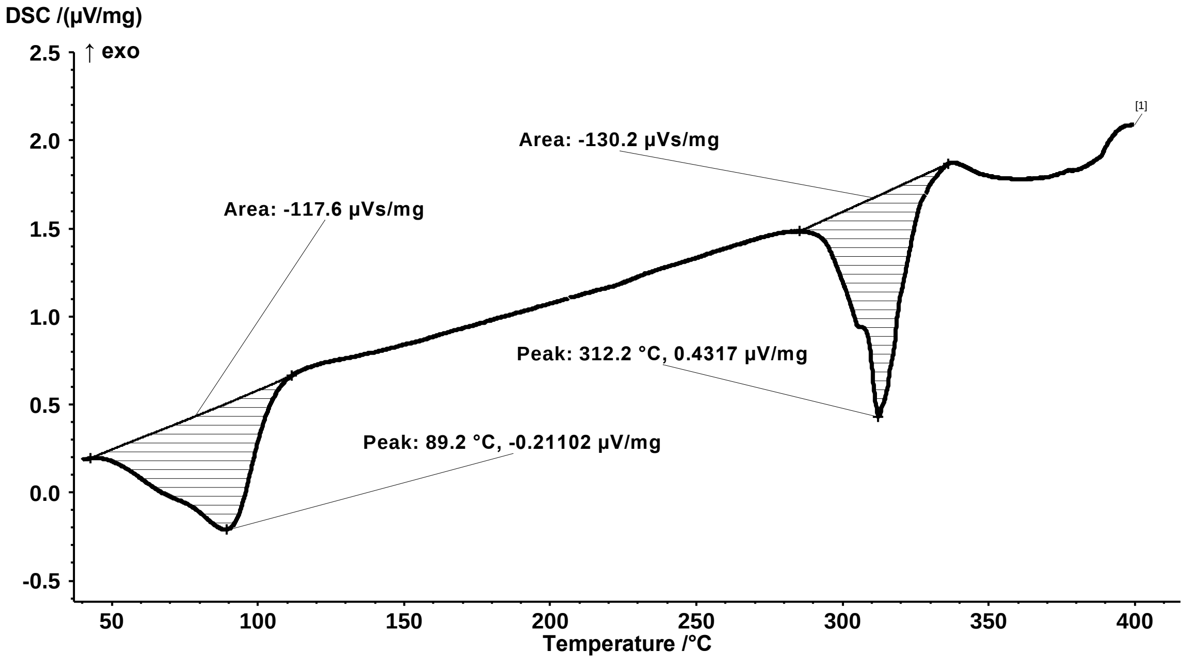

Figure S31: DSC analysis of *β-CD/ASO_3:1_b* complex.

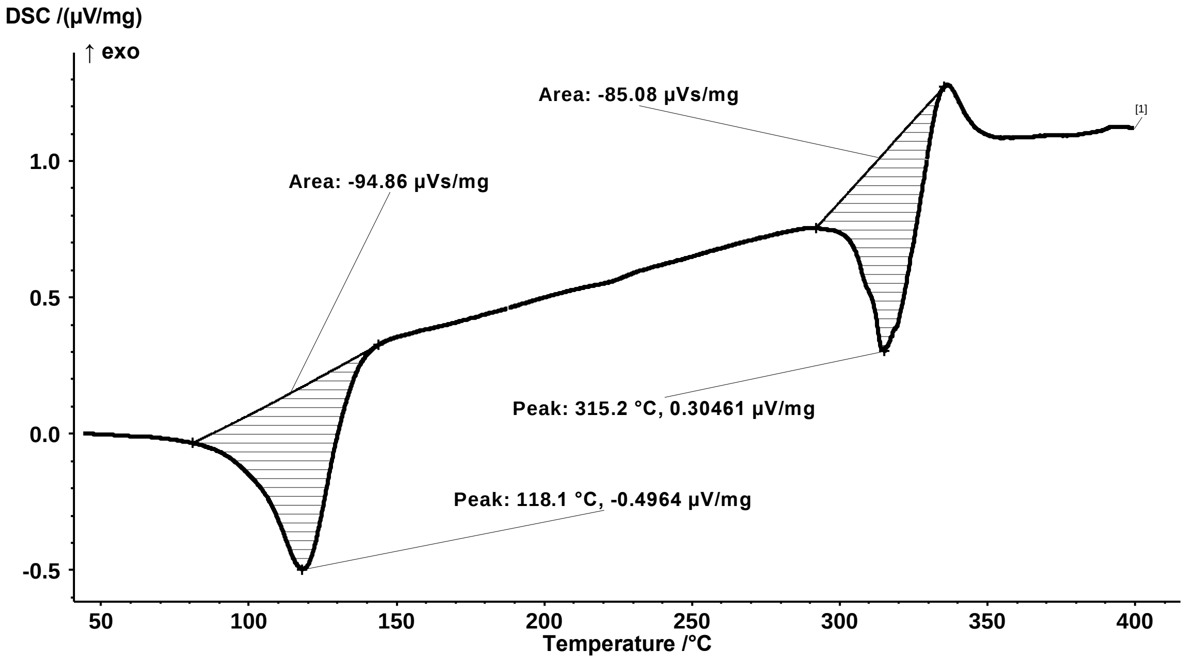

Figure S32: DSC analysis of *β-CD/ASO_1:1(k)_a* complex.

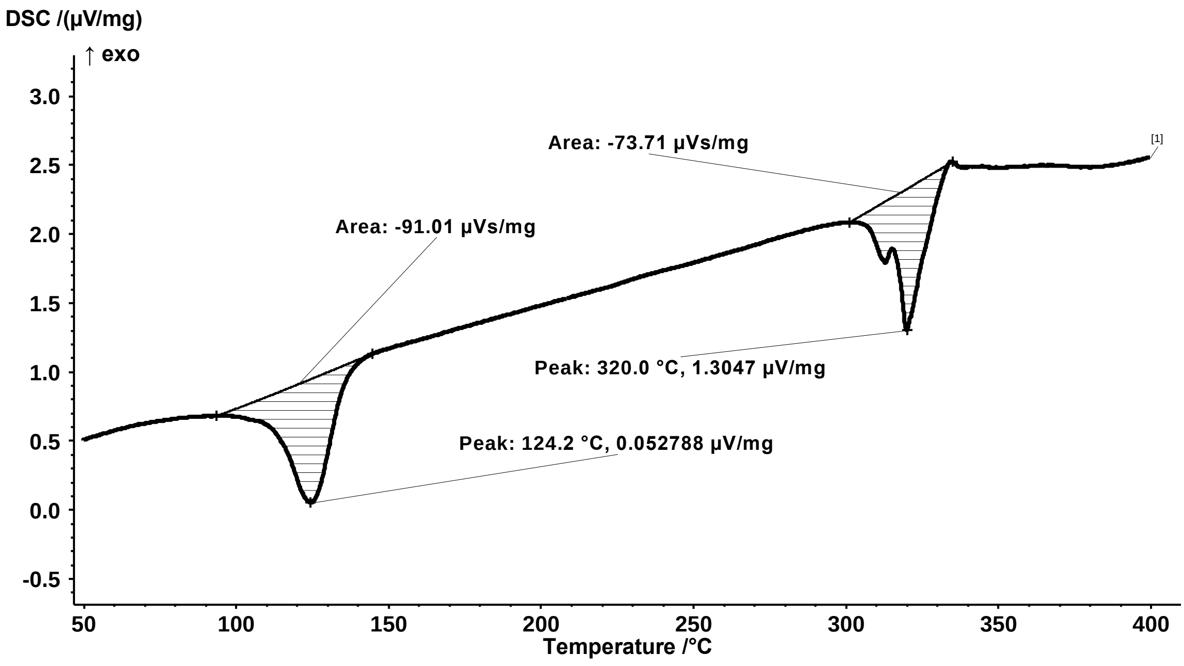

Figure S33: DSC analysis of *β-CD/ASO_1:1(k)_b* complex.

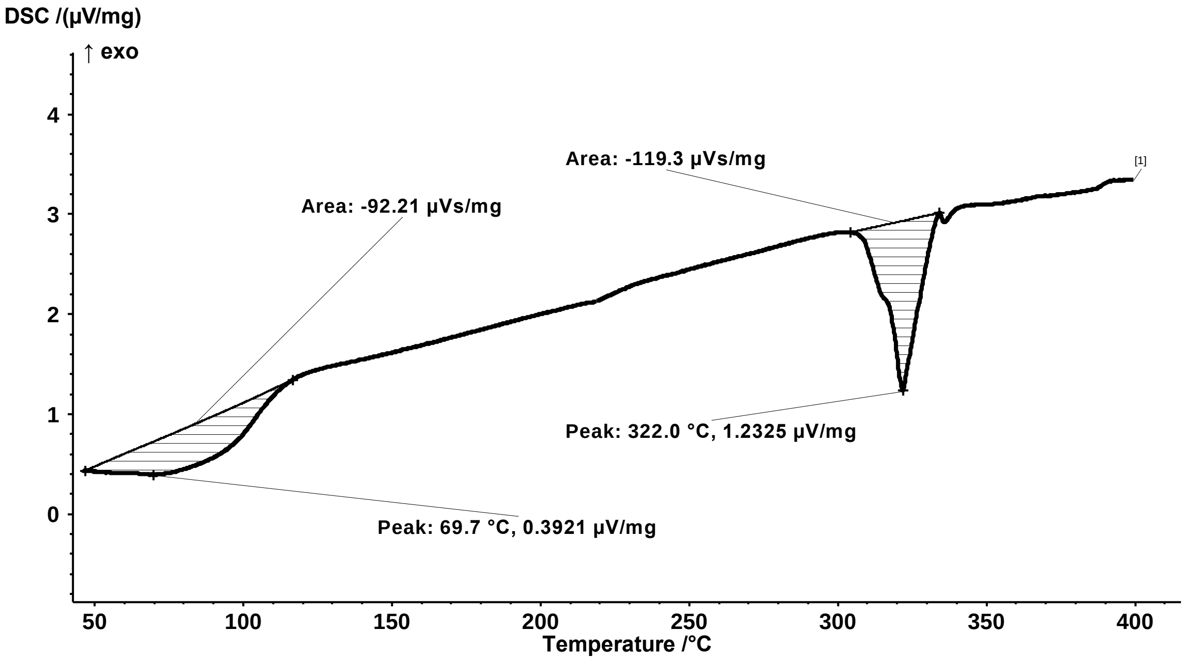

Figure S34: DSC analysis of *β-CD/ASO_3:1(k)_a* complex.

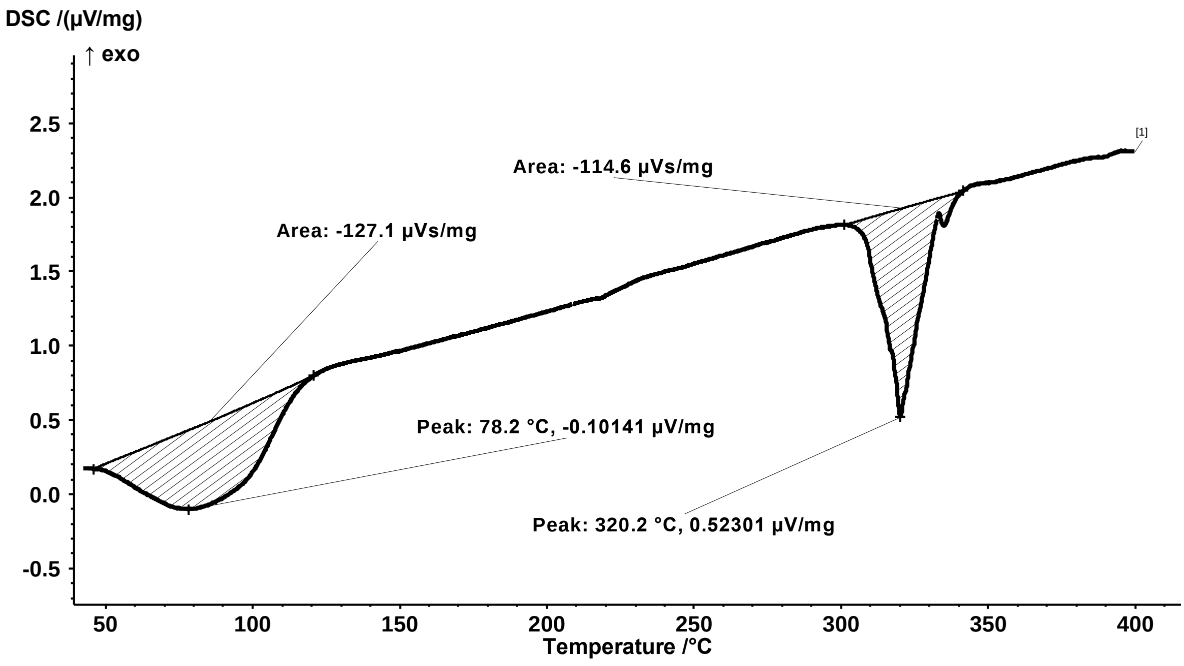

Figure S35: DSC analysis of *β-CD/ASO_3:1(k)_b* complex.

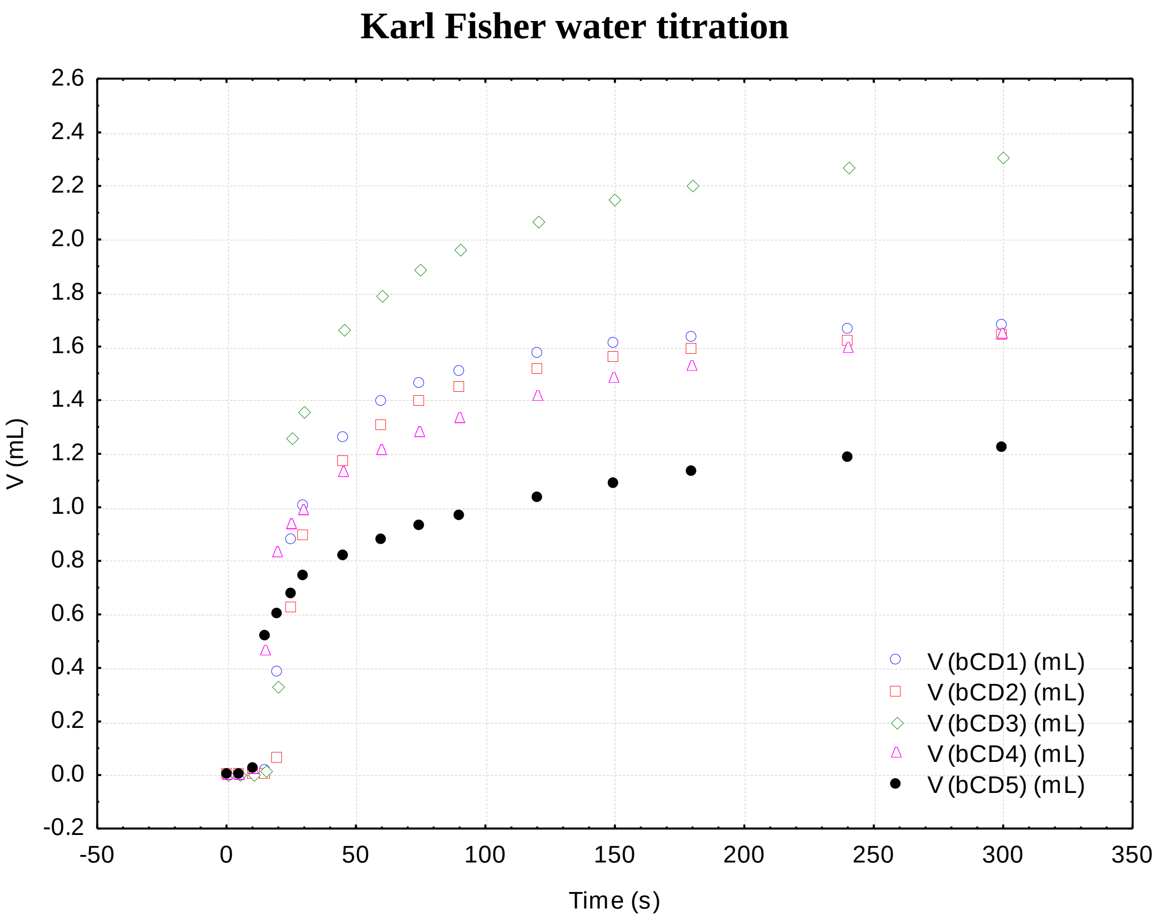

Figure S36: *Volume versus Time* plot from the KFT analysis of commercial β-cyclodextrin (*β-CD*).

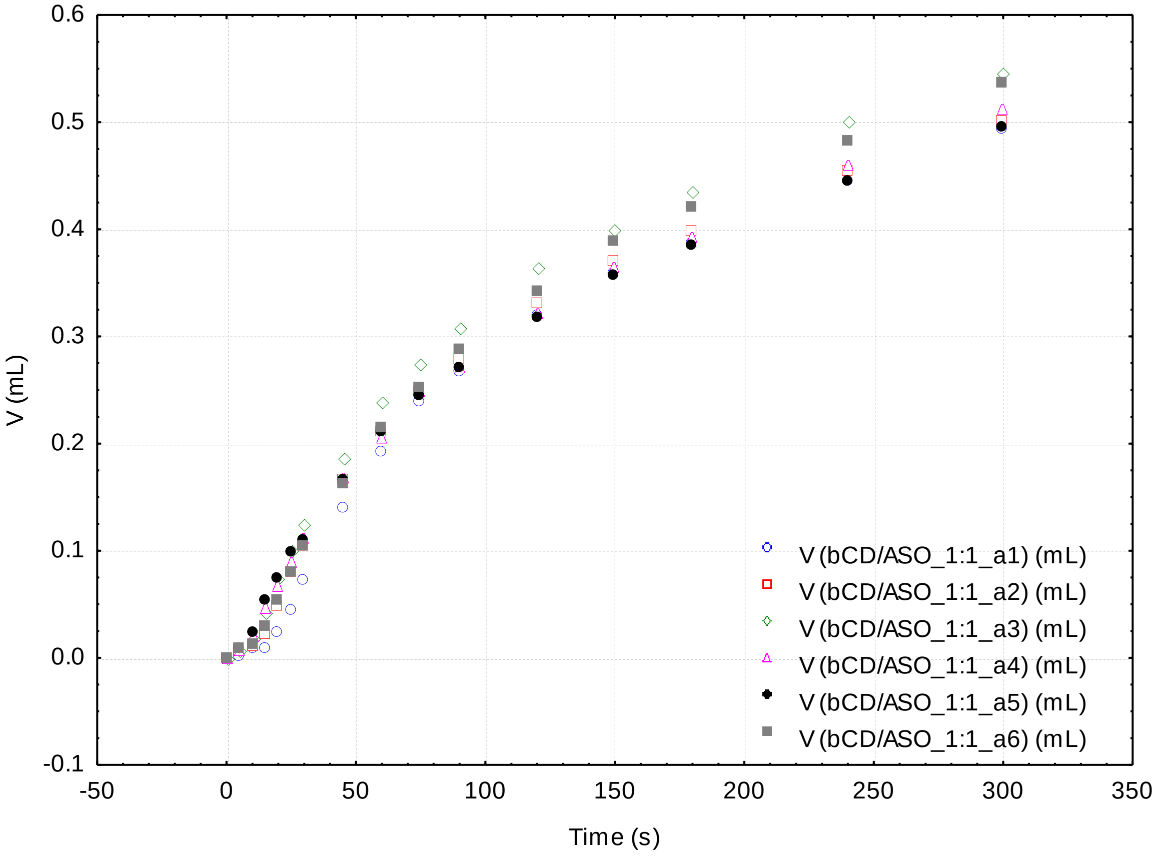

Figure S37: *Volume versus Time* plots from the KFT analysis of *β-CD/ASO_1:1_a* samples.

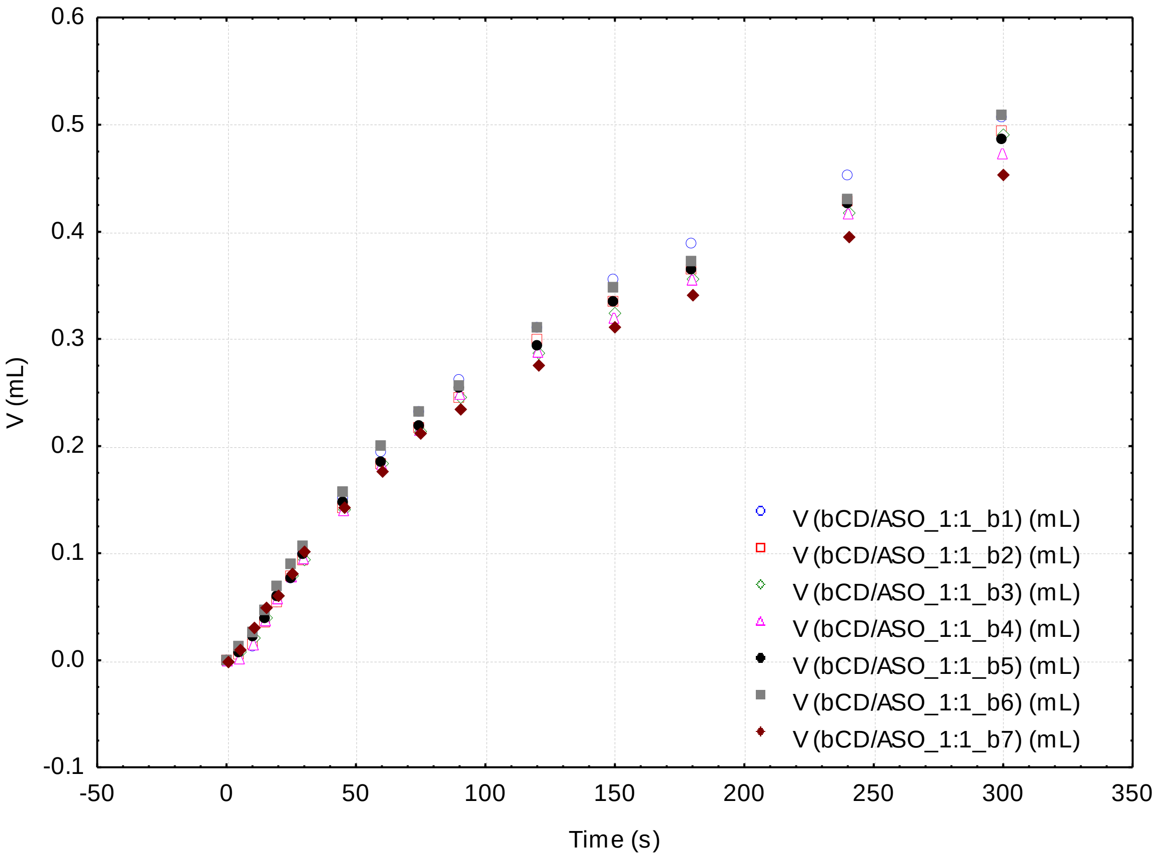

Figure S38: *Volume versus Time* plots from the KFT analysis of *β-CD/ASO_1:1_b* samples.

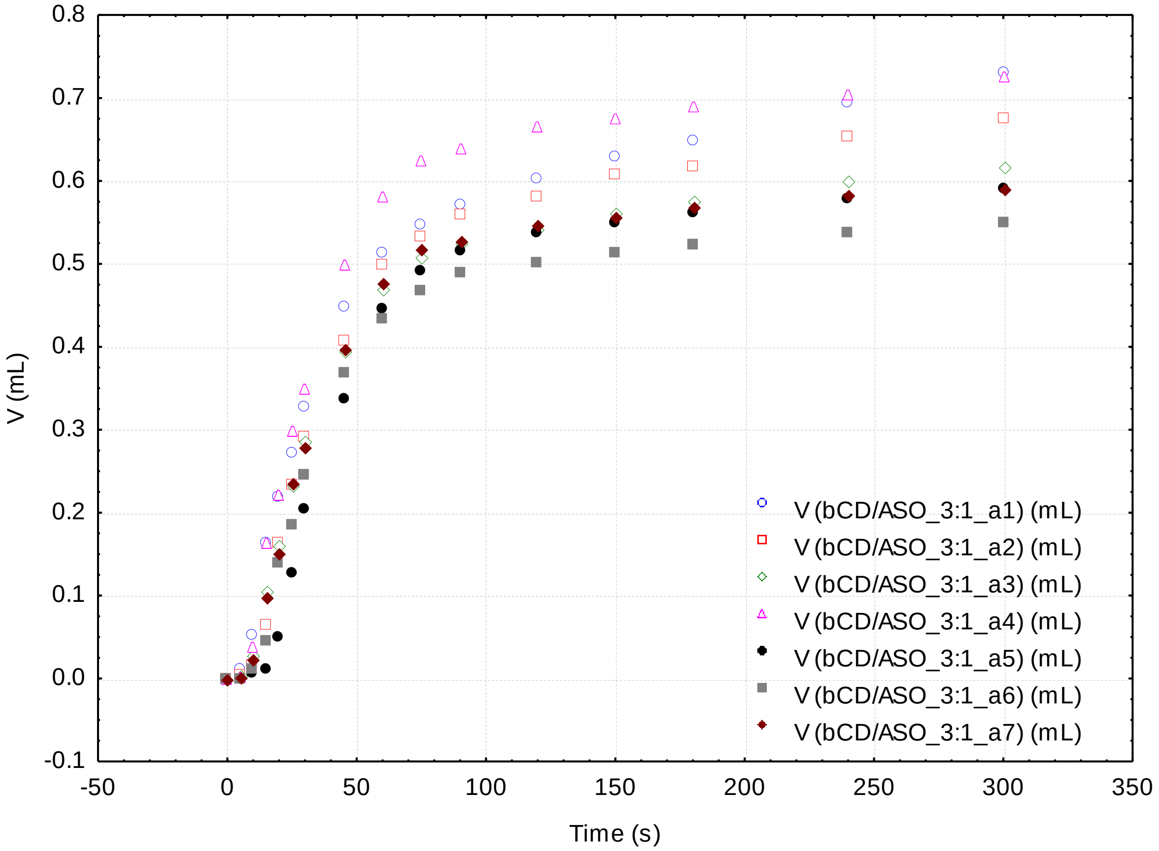

Figure S39: *Volume versus Time* plots from the KFT analysis of *β-CD/ASO_3:1_a* samples.

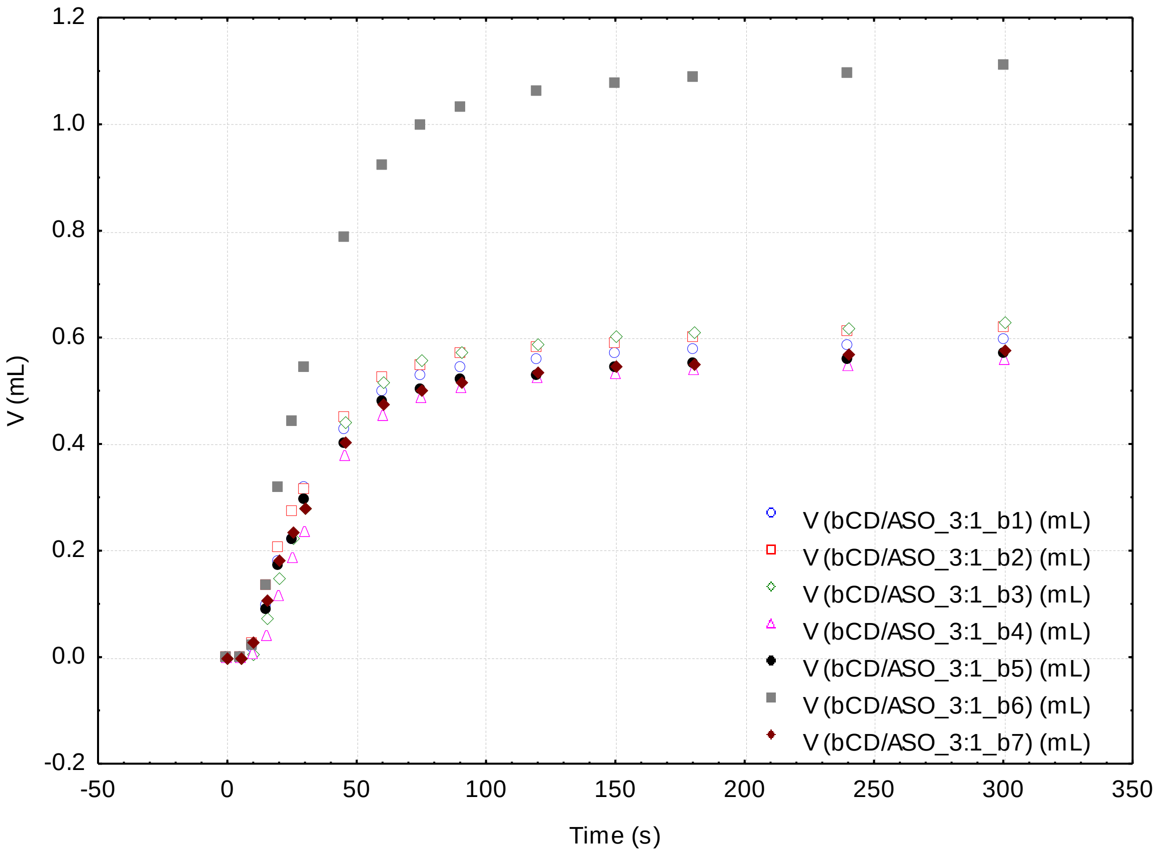

Figure S40: *Volume versus Time* plots from the KFT analysis of *β-CD/ASO_3:1_b* samples.

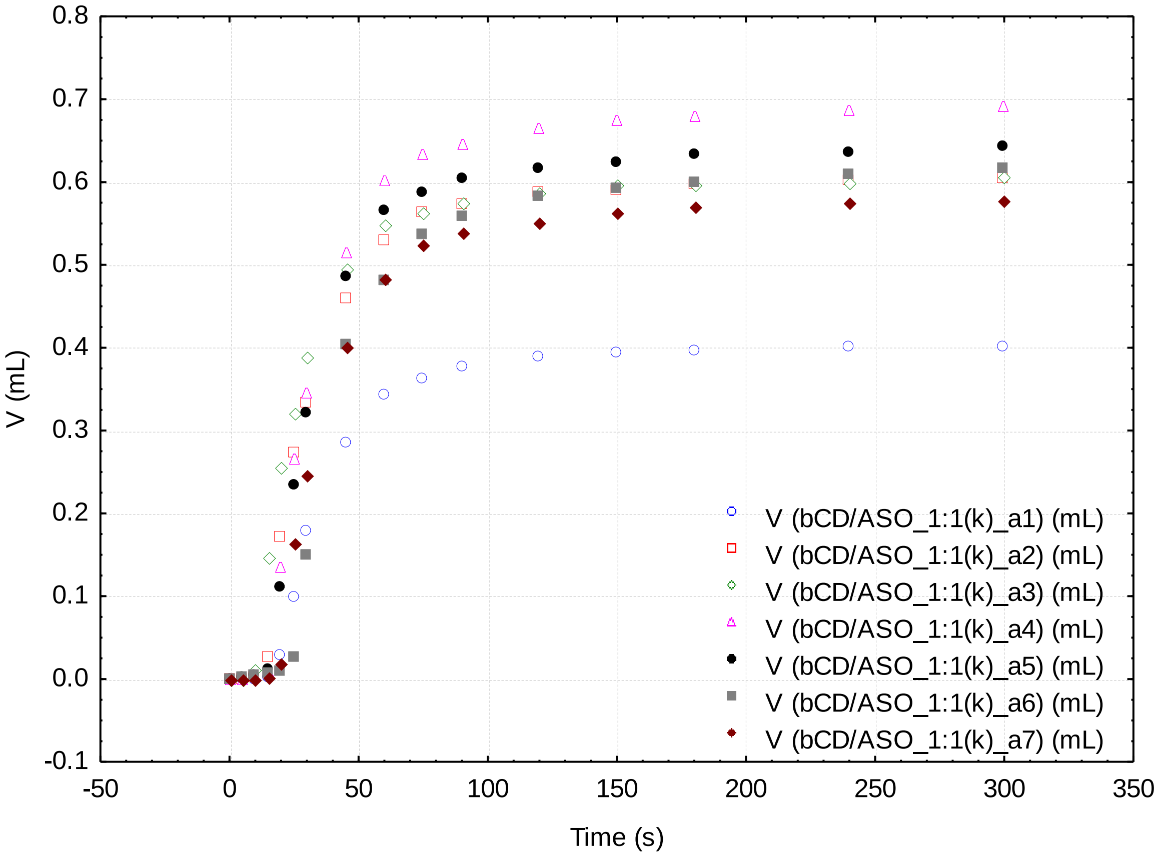

Figure S41: *Volume versus Time* plots from the KFT analysis of *β-CD/ASO_1:1(k)_a* samples.

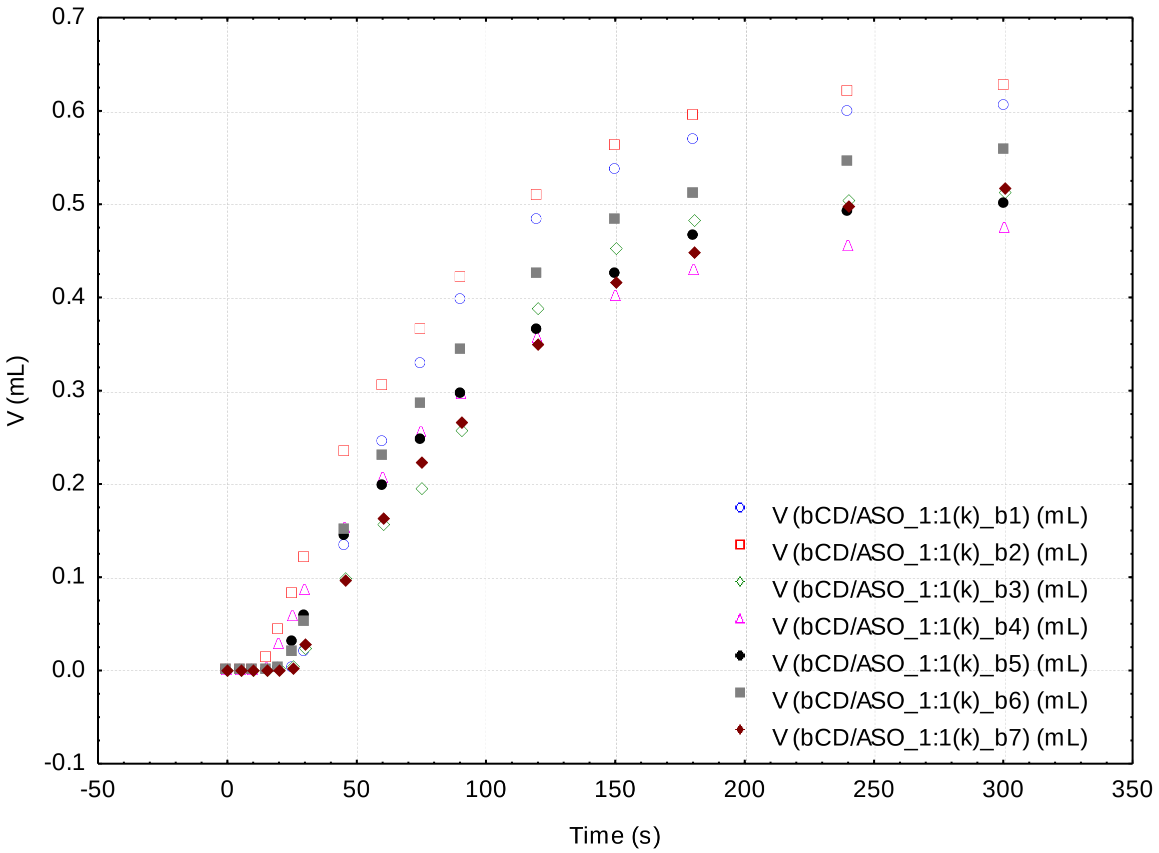

Figure S42: *Volume versus Time* plots from the KFT analysis of *β-CD/ASO_1:1(k)_b* samples.

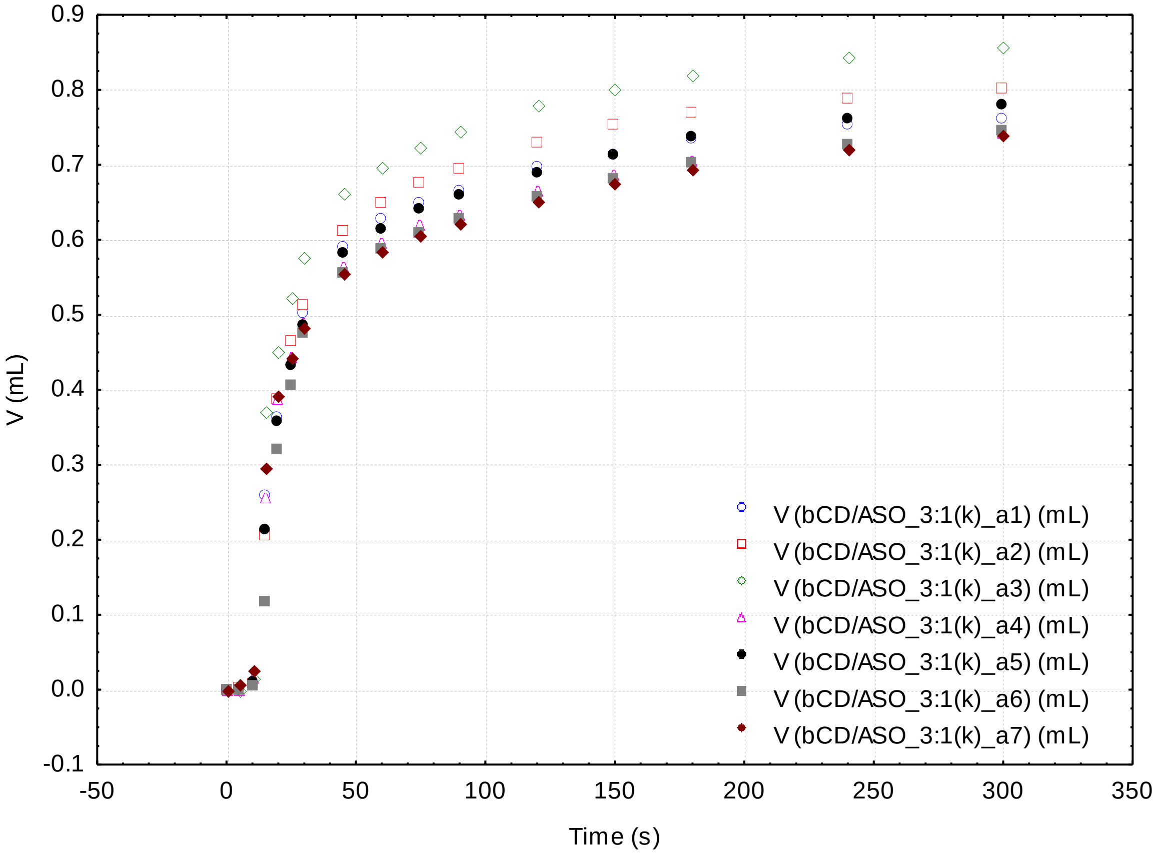

Figure S43: *Volume versus Time* plots from the KFT analysis of *β-CD/ASO_3:1(k)_a* samples.

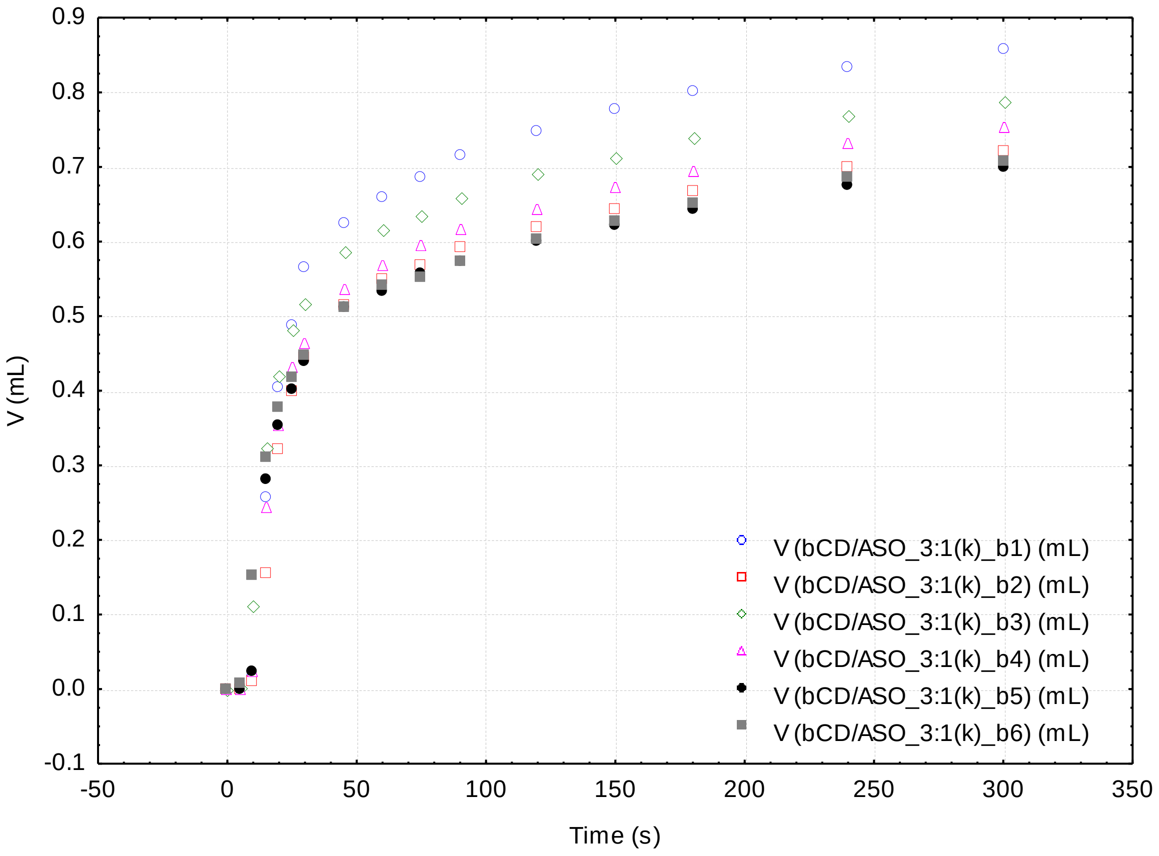

Figure S44: *Volume versus Time* plots from the KFT analysis of *β-CD/ASO_3:1(k)_b* samples.
